# Benefits of global mutant huntingtin lowering diminish over time in a Huntington’s disease mouse model

**DOI:** 10.1172/jci.insight.161769

**Published:** 2022-10-24

**Authors:** Deanna M. Marchionini, Jeh-Ping Liu, Alberto Ambesi-Impiombato, Kimberly Kerker, Kim Cirillo, Mukesh Bansal, Rich Mushlin, Daniela Brunner, Sylvie Ramboz, Mei Kwan, Kirsten Kuhlbrodt, Karsten Tillack, Finn Peters, Leena Rauhala, John Obenauer, Jonathan R. Greene, Christopher Hartl, Vinod Khetarpal, Brenda Lager, Jim Rosinski, Jeff Aaronson, Morshed Alam, Ethan Signer, Ignacio Muñoz-Sanjuán, David Howland, Scott O. Zeitlin

**Affiliations:** 1 CHDI Management/CHDI Foundation, New York, New York, USA.; 2 University of Virginia School of Medicine, Charlottesville, Virginia, USA.; 3 Psychogenics Inc., Paramus, New Jersey, USA.; 4 Evotec SE, Hamburg, Germany.; 5 Charles River, Kuopio, Finland.; 6 Rancho Biosciences, San Diego, California, USA.

**Keywords:** Neuroscience, Neurodegeneration

## Abstract

We have developed an inducible Huntington’s disease (HD) mouse model that allows temporal control of whole-body allele-specific mutant huntingtin (m*Htt*) expression. We asked whether moderate global lowering of m*Htt* (~50%) was sufficient for long-term amelioration of HD-related deficits and, if so, whether early m*Htt* lowering (before measurable deficits) was required. Both early and late m*Htt* lowering delayed behavioral dysfunction and mHTT protein aggregation, as measured biochemically. However, long-term follow-up revealed that the benefits, in all m*Htt-*lowering groups, attenuated by 12 months of age. While early m*Htt* lowering attenuated cortical and striatal transcriptional dysregulation evaluated at 6 months of age, the benefits diminished by 12 months of age, and late m*Htt* lowering did not ameliorate striatal transcriptional dysregulation at 12 months of age. Only early m*Htt* lowering delayed the elevation in cerebrospinal fluid neurofilament light chain that we observed in our model starting at 9 months of age. As small-molecule *HTT*-lowering therapeutics progress to the clinic, our findings suggest that moderate m*Htt* lowering allows disease progression to continue, albeit at a slower rate, and could be relevant to the degree of m*HTT* lowering required to sustain long-term benefits in humans.

## Introduction

Huntington’s disease (HD) is a progressive autosomal dominant neurodegenerative disorder that is caused by the expansion of a CAG repeat within the huntingtin (*HTT*) gene that encodes an expanded polyglutamine (polyQ) tract in the HTT protein. Although treatments delaying onset and progression of HD are not yet available, several *HTT*-lowering strategies are already under clinical evaluation. Phase 1b/2a–3 clinical trials testing the efficacy of antisense oligonucleotides (ASOs) targeting either total *HTT* expression (tominersen, Roche/Ionis: ClinicalTrials.gov; NCT03761849) or selective m*HTT* expression (WVE-120101 and -2, Wave Life Sciences: NCT03225833 and NCT03225846) were halted in March 2021 prior to reaching their planned end points (1), but other clinical trials using an AAV5-miHTT vector for *HTT* lowering delivered to the caudate and putamen are currently in phase 1/2a trials (AMT-130, uniQure; NCT04120493), and phase 1/2b trials of small-molecule *HTT*-splicing modulators that reduce *HTT* pre-mRNA levels are either underway (PTC Therapeutics; PTC518) or will soon begin (Novartis, LMI070; branaplam).

To complement clinical trials, animal models can be used to address several critical questions that remain unanswered about *HTT*-lowering therapeutic strategies. First, it is currently unclear which HTT species and how much HTT need to be lowered. It is not known whether m*HTT* needs to be selectively lowered (as opposed to total *HTT*) since HTT protein has roles in multiple biological processes and loss of WT HTT function may counterbalance the benefits of m*HTT* lowering or induce safety liabilities. KO of *Htt* in the mouse is embryonic lethal, and conditional KO of *Htt* in adult mice can produce gross anatomical disturbances in the periphery (2, 3). Furthermore, not all clinical candidates lower the alternatively processed *HTT1a* transcript, which encodes a pathogenic protein (4). Importantly, it is unclear how much m*HTT* lowering is required to slow disease progression. Previous studies with limited brain distribution of total *Htt* or allele-specific m*Htt* lowering greater than 50% have shown attenuation of discrete phenotypes in HD rodent models (5–17).

Second, it is unknown whether therapeutic benefit requires m*HTT* suppression throughout the nervous system or the entire body, or if lowering in specific CNS circuits will suffice for maintaining motor, cognitive, and psychiatric function, as individuals with HD present with peripheral comorbidities (18) and HD mouse models display peripheral alterations (19–21) that might not improve following nervous system lowering of m*HTT*. Cre-mediated excision of m*HTT* in both striatal and cortical neurons in the bacterial artificial chromosome transgenic HD mouse model (BACHD) showed significant preservation of striatal synaptic function, behavioral readouts, and brain volume compared with BACHD mice. In contrast, Cre excision of m*HTT* in either striatal or cortical neurons alone only partially preserved function in the BACHD mice (16). Although region-specific lowering of m*HTT* is expected to benefit cell-autonomous neuronal function, functional improvements will likely require targeting more than just the striatum.

Third, it is unclear if there is a critical time period during which *HTT* lowering should begin and how much m*HTT* lowering is required to slow disease progression long-term. To date, HD clinical trials with *HTT*-lowering therapeutics have used symptomatic participants, although changes in the brains of individuals with HD prior to the appearance of symptoms have been documented (22–27). In addition, embryonic cortical development is altered by m*HTT* expression in both HD–knock-in mouse models and humans (17, 28–30). Thus, it will be important to determine the disease stage at which *HTT*-lowering interventions need to begin and to define biomarkers that respond to *HTT-*lowering therapeutic agents. While it is difficult to compare different *HTT*-lowering interventions due to differing mechanisms of action, off-target effects, regional distribution patterns, and differential *HTT* lowering in individual cells, it is not yet known whether long-term moderate reduction in m*HTT* expression levels (~40%–50%, similar to the lowering achieved by HTT-lowering modalities currently in clinical trials) will be sufficient to sustain long-term phenotypic benefits in aged animals (5–17).

To begin to address these questions, we generated an inducible HD–knock-in mouse model, LacQ140, in which the expression of m*Htt* can be regulated by adding or withdrawing the lactose analog isopropyl β-D-1-thiogalactopyranoside (IPTG) in their drinking water or chow, using an established *Lac* operator/repressor system (31, 32). We found that early m*Htt* lowering greatly delayed formation of mHTT protein inclusion bodies (IBs), ameliorated behavioral and transcriptional dysregulation, and delayed the increase of neurofilament light chain (NFL) levels in cerebrospinal fluid (CSF) that the model exhibited at 9 month of age. However, all of these benefits were attenuated by 12 months of age. Late m*Htt* lowering did not improve transcriptional dysregulation, and behavioral benefits were diminished at 12 months of age, suggesting that earlier m*Htt* lowering would be more beneficial.

## Results

### Characterization of the LacQ140 HD model.

**Figure 1:**
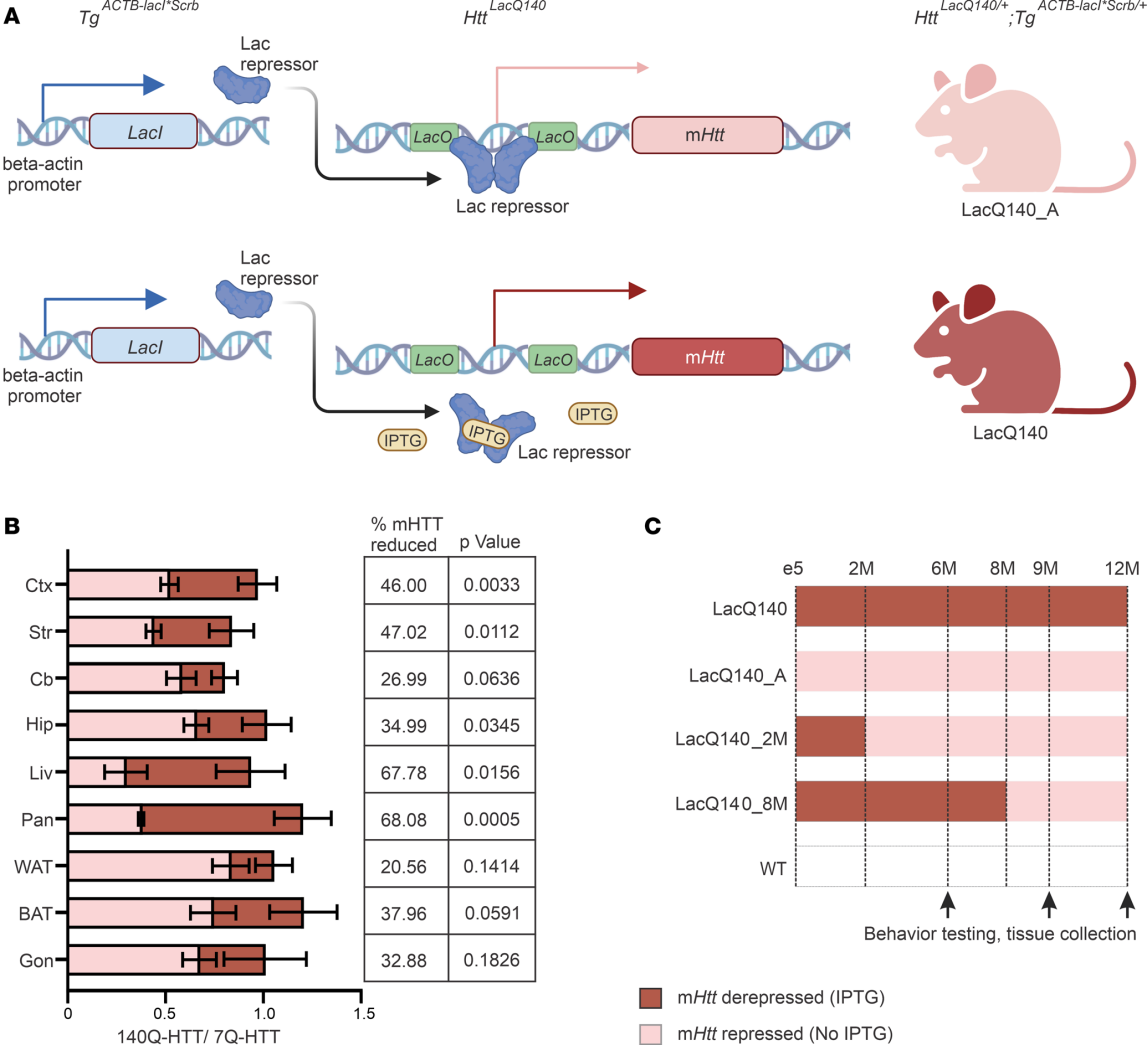
Outline of experimental paradigm. (**A**) Schematic of the Tg^ACTB-lacI*Scrb^ transgene and the *Htt^LacQ140^* allele. The default state of the *Htt^LacQ140/+^*; Tg^ACTB-lacI*Scrb/+^ mouse is global repression of m*Htt* due to Lac repressor binding to the Lac operators (LacQ140_A). The addition of IPTG interrupts the binding between the Lac repressors and operators, resulting in a derepressed state and maximal expression of m*Htt* (LacQ140). Schematic created with BioRender. (**B**) Repression of m*Htt* in different tissues 25 days after IPTG withdrawal at 6 months of age. LacQ140 mice were continuously provided with IPTG in their drinking water until they reached 6 months of age. The 140Q-HTT/7Q-HTT ratio on the last day of IPTG treatment (red) and on the 25th day after IPTG withdrawn (pink) was quantified by Western blot (MAB2166) using protein extracts isolated from cortex (Ctx), striatum (Str), cerebellum (Cb), hippocampus (Hip), liver (Liv), pancreas (Pan), white adipose tissue (WAT), brown adipose tissue (BAT), and gonad (testis and ovary, Gon). *n* = 5/group (3 males and 2 females); data are shown as mean ± SEM % of mHTT reduction, and *P* values from unpaired *t* tests for each tissue are shown. (**C**) An outline of the mice used in this study. LacQ140 treated with IPTG continuously starting from E5 served as the HD mouse model. m*Htt* was always lowered (LacQ140_A), lowered starting at 2 months of age by IPTG removal (LacQ140_2M), or lowered starting at 8 months of age by IPTG removal (LacQ140_8M). Littermate WT mice (Tg^ACTB-lacI*Scrb/+^) were treated with or without IPTG for the course of this study. Red shaded area indicates maximal expression of m*Htt* during IPTG treatment; pink shaded area indicates m*Htt* lowering without IPTG. Mice were examined for behavior and tissue collection at 6, 9, and 12 months of age.

To generate a *LacO*/*LacIR*-regulatable HD mouse model, we first inserted *Lac* operator sequences within the *Htt* promoter flanking the transcriptional start site in our Q140-Htt HD mouse model (Supplemental Figure 1A; supplemental material available online with this article; https://doi.org/10.1172/jci.insight.161769DS1). We then crossed these mice with a transgenic line, Tg^ACTB-lacI*Scrb^ (31), ubiquitously expressing a modified version of the *E*. *coli* *Lac* repressor gene to obtain *Htt^LacQ140/+^* LacIR (LacQ140) progeny. The default state of the LacQ140 mouse is global repression of m*Htt* due to *Lac* repressor binding to the *Lac* operators. The continuous administration of IPTG starting from E5 interrupts the binding between the *Lac* repressor and operators, resulting in a derepressed state and in maximal expression of m*Htt* in LacQ140 that is comparable with the levels of m*Htt* expression in *Htt^LacQ140^* and *Htt^140Q^* mice (Figure 1A and Supplemental Figure 1, B–D).

To evaluate the kinetics of m*Htt* lowering, brain and peripheral tissues were collected for RNA and protein analyses from 6-month-old LacQ140 mice at 0, 3, 6, 10, 15, 20, and 25 days following IPTG withdrawal. Maximal repression of m*Htt* mRNA levels was attained within 3 days following IPTG withdrawal (Supplemental Figure 1B), while maximal mHTT protein reduction was observed 15 days following IPTG withdrawal (Supplemental Figure 1C). By 25 days after IPTG withdrawal, a reduction in mHTT in the cortex (46%), striatum (47%), cerebellum (27%), hippocampus (35%), liver (68%), pancreas (68%), white adipose tissue (21%), brown adipose tissue (38%), and gonads (33%) was observed (Figure 1B). These results demonstrate that moderate global mHTT lowering can be achieved ~2 weeks after IPTG withdrawal in this mouse model.

### Consistent repression of mHtt in all ages examined.

We confirmed that the *Lac* operator–repressor system was functional and responsive to IPTG for the duration of our experiments. We monitored the expression of mHTT protein in the cerebellum, a brain region with low levels of mHTT aggregation, obtained from 6- to 12-month-old mice by MSD assay. Maximum derepression was achieved with IPTG treatment and resulted in equal and full expression of mHTT in the LacQ140 mice at 6, 9, and 12 months, demonstrating that IPTG was able to disrupt repressor-operator binding regardless of age. Mice with no IPTG (LacQ140_A) or IPTG removed at 2 or 8 months of age (LacQ140_2M or LacQ140_8M) exhibited a similar reduction in mHTT protein at all ages examined, demonstrating that operator-repressor binding was consistent throughout age (Supplemental Figure 2A).

We also examined whether there were sex differences in the extent of m*Htt* lowering by evaluating m*Htt* transcript levels in the cerebellum from 6- to 12-month-old mice with early and late m*Htt* lowering using quantitative PCR (qPCR). We found that the *Lac* repressor performed equally well in lowering m*Htt* expression in both sexes at all ages examined (Supplemental Figure 2B).

### Htt1a transcript was lowered following IPTG withdrawal.

To examine whether the *Htt1a* transcript levels were also lowered following IPTG withdrawal, we first confirmed that, at 6 months of age, *Htt1a* transcript was present in the cortex of LacQ140 and not in WT mice, as measured by probes to intron 1 before the first cryptic poly(A) site (*I*_1_-*pA*_1_) and to intron 1 sequences after this but before the second cryptic poly(A) site (*I*_1_-*pA*_2_) (33). *Htt1a* transcript levels were significantly reduced in the cortex of LacQ140_2M mice. As a control for contaminating Htt pre-mRNA, there were no differences in levels of incompletely spliced intron 1 transcripts that did not terminate at cryptic poly(A) sites in any groups, as measured by probes to the 3′ end of intro 1 (I_1_-3′ ) and within intron 3 (I_3_) (Supplemental Figure 3).

### Early mHtt lowering ameliorated high-content behavioral deficits.

To examine the effects of early versus late m*Htt* lowering on LacQ140 HD mouse model phenotypes, cohorts of mice with early m*Htt* lowering (LacQ140_A, lowering from conception; LacQ140_2M, lowering started at 2 months of age) or late m*Htt-*lowering LacQ140_8M, lowering started at 8 months of age), and WT controls (Tg^ACTB-lacI*Scrb/+^ [with or without continuous IPTG treatment starting at E5]) were followed until 12 months of age (Figure 1C).

**Figure 2:**
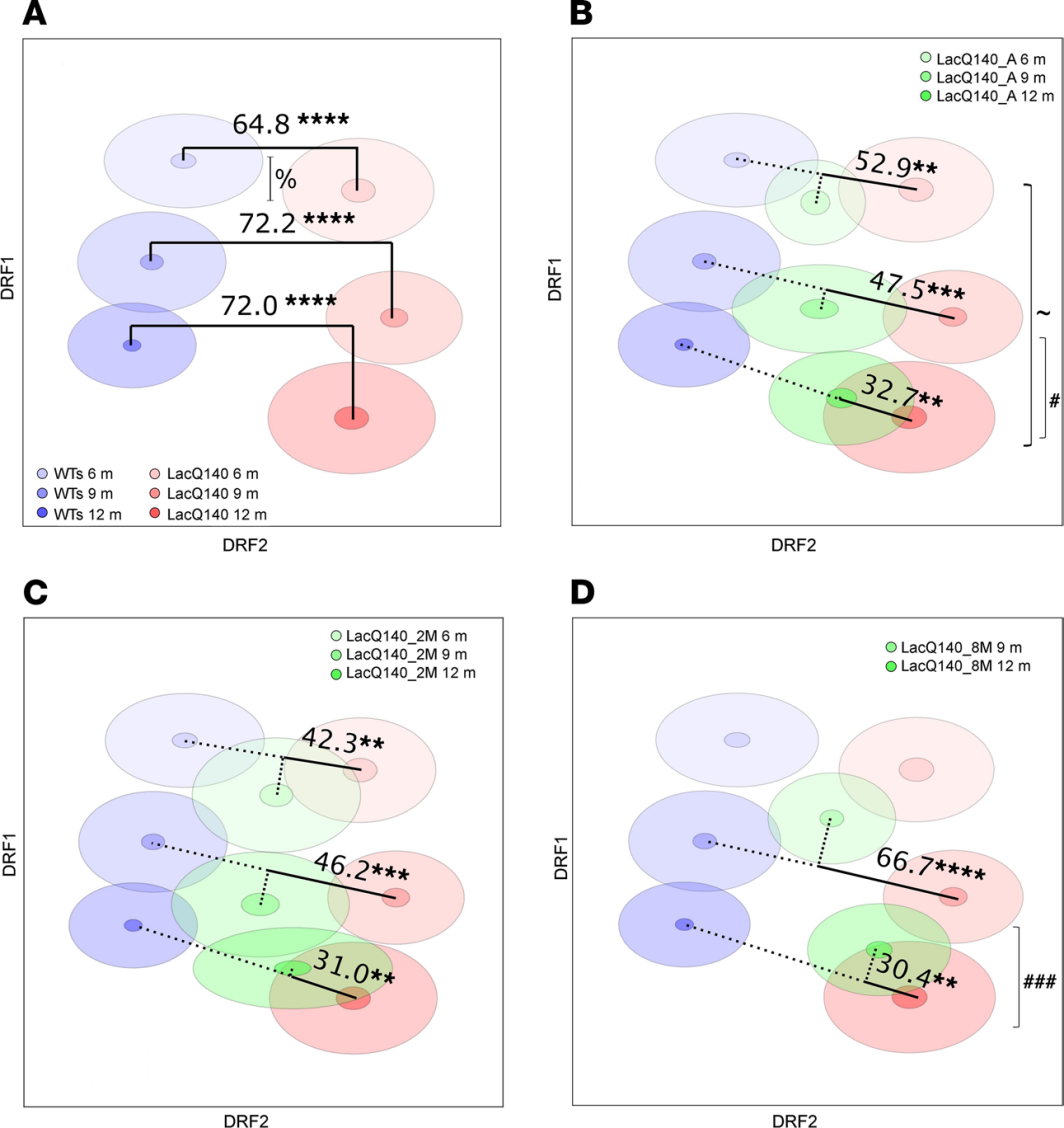
Behavioral phenotypes were delayed by m*Htt* lowering. Decorrelated ranked feature analysis for WTs and LacQ140 groups at different ages and effect of m*Htt* lowering. (**A**) Blue clouds represent WTs groups (WT: *n* = 8 males, *n* = 16 females; WT + IPTG: *n* = 15 males, *n* = 16 females), red clouds represent LacQ140 groups (*n* = 14 males, *n* = 16 females). The center of each cloud represents the mean, small darker shaded ellipses represent the SEM, and the larger lighter shaded ellipses represent the SD of each groups’ distribution. Discrimination Indices between pairs of WTs and LacQ140 groups at each age capture the strength of the phenotypic signature of this model. (**B**–**D**) Each m*Htt*-lowered group was projected onto the DRF space to quantify the m*Htt*-lowering effect of LacQ140_A (*n* = 8 males, *n* = 15 females) (**B**), LacQ140_2M (*n* = 16 males, *n* = 16 females) (**C**), and LacQ140_8M (*n* = 16 males, *n* = 16 females) (**D**), compared with LacQ140 (solid line). ****P < 0.0001, ***P < 0.001, **P < 0.01. ^%^*Z* = 2.6, *P* = 0.005 (1-tailed *Z* test). An additional mixed-model analysis evaluated the effects of age and treatment (different m*Htt*-lowering regimens) as factors and their interaction. Main effects of age and its interaction with treatment were significant (F_[2,89]_ = 16.02, *P* < 0.0001, and F_[3, 90.8]_, *P* = 0.038, respectively) but the treatment main effect was not (F_[2, 87.8]_ = 0.44, *P* = 0.64). ^~^*P* = 0.03, ^#^*P* = 0.01, ^###^*P* < 0.0001.

Unbiased high-content behavioral profiling with SmartCube, NeuroCube, and PhenoCube (34–37) platforms was used to evaluate the consequences of m*Htt* lowering on HD mouse model behavioral deficits. We first calculated the overall discrimination between the WTs and LacQ140 groups by testing the 6 groups within the same decorrelated ranked feature analysis (DRFA). This 6-group DRF space captures differences due to age and to phenotype and its progression; it, therefore, can be interpreted as providing “aging” and “disease” orthogonal axes (Figure 2A). We observed a robust discrimination between the LacQ140 groups and WT groups in a 6-group cloud analysis, where the 3 test ages were considered in the same analysis. At 6, 9, and 12 months of age, there was a 64.8%, 72.2%, and 72.0% difference, respectively, in features between WT and LacQ140 mice, demonstrating that this HD mouse model has measurable behavioral dysfunction by 6 months of age. This dimensionality reduction approach clearly separated age and disease effects along approximately orthogonal axes, and this separation was consistent with disease progression across test ages, as the age of each experimental group progressed along 1 axis. There was significant disease progression between 6 and 9 months of age that was lost between 9 and 12 months (Figure 2A).

The 6-group space allows for testing the effect of m*Htt*-lowering manipulation in terms of disease and age axes, by projecting each of the m*Htt*-lowered groups into this space. At 6 months of age, the LacQ140 and LacQ140_A groups showed a discrimination of 52.9%. The difference between LacQ140 and LacQ140_A groups was somewhat lower at the older ages (47.5% and 32.7% at 9 and 12 months of age, respectively; Figure 2B). The discriminations between the LacQ140 and LacQ140_2M groups were 42.3%, 46.2%, and 31.0% at 6, 9, and 12 months of age, respectively (Figure 2C). The discriminations between the LacQ140 and LacQ140_8M groups were 66.7% and 30.4% at 9 and 12 months of age, respectively (Figure 2D). Of note, the LacQ140_8M group at 9 months of age appeared relatively less aligned with the other 2 corresponding target and reference clouds at 9 months of age (Figure 2); this could reflect the confounding effect of 1 less exposure to the cube testing for this experimental group. Further statistical analyses were done to understand if the discrimination between the m*Htt*-lowered groups and the LacQ140 declined with age (Figure 2). While early 2-month m*Htt* lowering did not reveal a significant decline in behavioral discrimination throughout age, always-lowered and late m*Htt* lowering, revealed a significant reduction in discrimination from the LacQ140 group between 9 and 12 months of age, demonstrating that the behavioral deficits were delayed but not halted with *mHtt* lowering. Top features are presented in Supplemental Table 1; we selected 1 feature to plot as an example. The LacQ140 mice exhibited increased immobility at 6 and 12 months of age. Early m*Htt* lowering increased mobility, compared to LacQ140, at 6 months of age only (Supplemental Figure 4).

### mHtt lowering delayed mHTT protein aggregation.

To examine the effect of m*Htt* lowering on mHTT protein aggregate formation, different brain regions were isolated from all groups of mice at 6, 9, and 12 months of age. Soluble mHTT protein was detected using the antibody pair 2B7-MW1 in an MSD platform, while mHTT aggregation was detected with the antibody pair MW8-4C9 (38).

**Figure 3:**
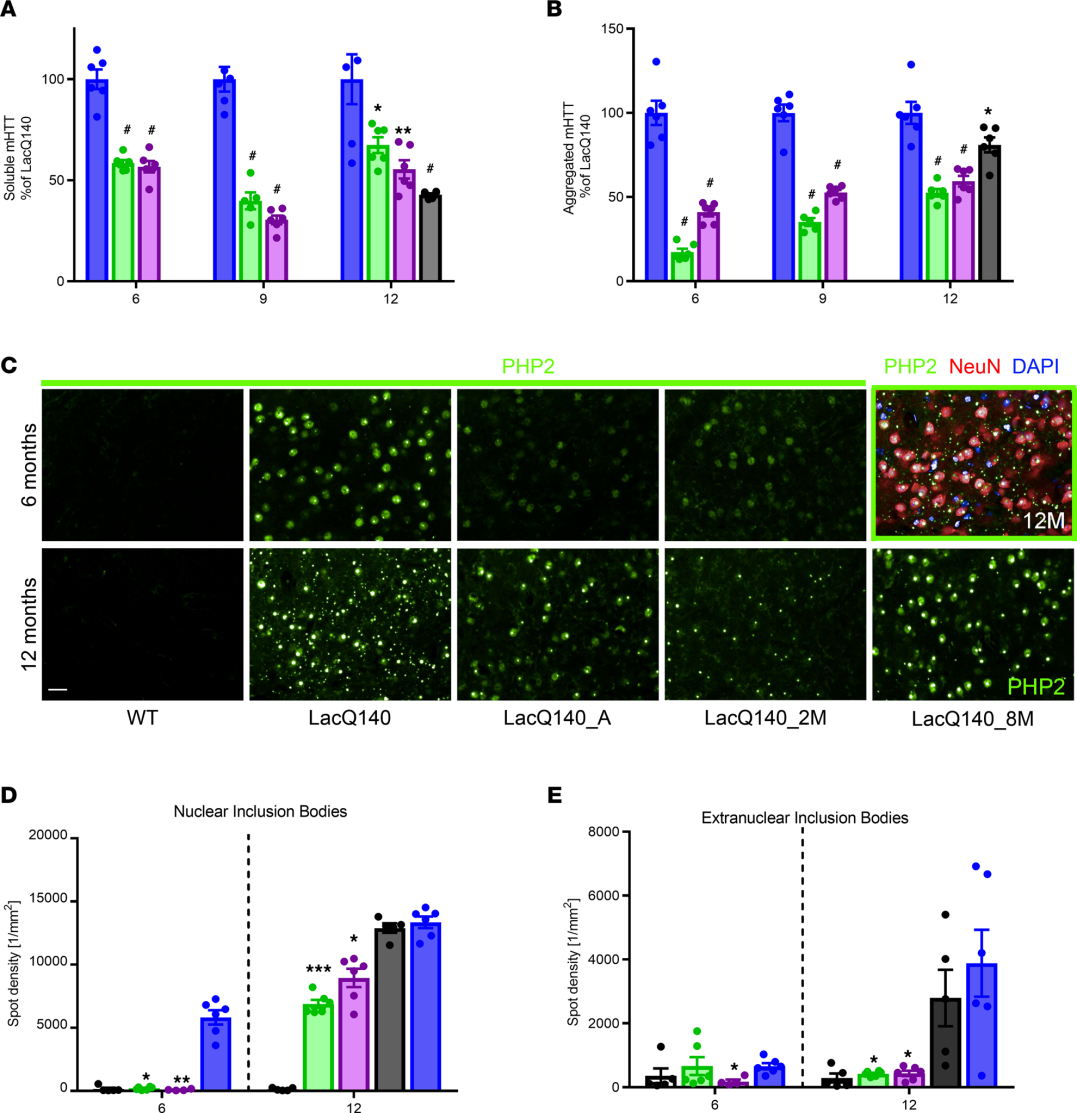
mHTT protein in the striatum. WT (white), LacQ140 (blue), LacQ140_A (green), and LacQ140_2M (purple) were sacrificed at 6, 9 and 12 months of age, while LacQ140_8M (black) were sacrificed at 12 months of age. (**A**) Soluble mHTT was measured in the striatum using 2B7-MW1 MSD. Data were normalized to LacQ140, set at 100% for each age. One-way ANOVA, followed by Bonferroni’s multiple-comparison test; ^#^*P* < 0.0001, **P < 0.0005, *P < 0.01. (**B**) Aggregated mHTT was measured in the striatum by MW8-4C9 MSD. Data were normalized to LacQ140, set at 100% for each age. One-way ANOVA, followed by Bonferroni’s multiple-comparison test; ^#^*P* < 0.0001, *P < 0.05. *n* = 6/group, except LacQ140_A at 9M *n* = 5; data are shown as mean ± SEM (**A** and **B)**. (**C**) Representative PHP2 immunolabeling of the striatum at 6 and 12 months. Scale bar: 20 μm. Green outlined panel with mHTT (PHP2; green), neurons (NeuN; red), and nucleus (DAPI; blue) is the same 12-month-old LacQ140 image used in the PHP2-only panel. (**D** and **E**) Nuclear (**D**) and extranuclear (**E**) quantitation of PHP2-ir spot density per mm^2^ in the striatum. Kruskal-Wallis, followed by Dunn’s multiple-comparison test, was performed separately at each age and compared each group with LacQ140. *P < 0.05, **P < 0.005, ***P < 0.001; LacQ140 (*n* = 6), LacQ140_A (*n* = 6), LacQ140_2M (*n* = 4 at 6 months and *n* = 6 at 12 months), LacQ140_8M (*n* = 5), WT (*n* = 5); data are shown as mean ± SEM. WT group was not included in statistical analysis.

In the striatum, all m*Htt*-lowered mouse groups exhibited significantly less (33%–70%) soluble mHTT, compared with the LacQ140 at each time point (Figure 3A). mHTT aggregation was reduced by 59%–83% in mice with early m*Htt* lowering at 6 months of age and was still reduced by 41%–47% at 12 months of age, suggesting a delay but not a halt in aggregation with moderate m*Htt* lowering. Late m*Htt* lowering also reduced striatal mHTT aggregation by 19% at 12 months (Figure 3B). Since the MSD assay likely cannot detect large mHTT IBs, we further characterized mHTT aggregation by IHC using the PHP2 antibody (39) on striatal sections (Figure 3C). At 6 months of age, LacQ140 mouse striatum exhibited strong diffuse nuclear PHP2 immunoreactivity (PHP2-ir), as well as nuclear and extranuclear IBs, while low levels of diffuse nuclear PHP2 staining were detected in some striatal neurons in early m*Htt*-lowering groups. By 12 months of age, large nuclear IBs and numerous extranuclear IBs were found in the LacQ140 striatal neurons, while fewer nuclear IBs were detected in the neurons of early m*Htt*-lowered groups. Quantification revealed a significant delay in formation of nuclear and extranuclear mHTT IBs with early m*Htt* lowering (33%–45% less nuclear IBs and 89% less extranuclear IBs compared with the LacQ140 mice); in contrast, late lowering did not result in a significant reduction in striatal IBs (Figure 3, D and E).

We also examined the effect of m*Htt* lowering on mHTT aggregate formation in the cortex and thalamus and found a significant reduction in mHTT aggregation in both early and late m*Htt*-lowered mice using the MSD assay. Compared with the striatum, a more substantial reduction in mHTT aggregation was detected in the cortex (79%–91% reduction in the early m*Htt*-lowering groups and a 70% reduction in the late m*Htt-*lowering group) and in the thalamus (53%–67% reduction in all m*Htt*-lowering groups; Supplemental Figure 5). IHC examination using the PHP2 antibody revealed a very slow progression in mHTT IB accumulation in the cortex, CA1 region of the hippocampus, and thalamus, even at 12 months of age, demonstrating that these brain regions exhibit much slower kinetics in mHTT aggregation, compared with the striatum. Few, if any, IBs were detected in these brain regions in the early m*Htt*-lowering groups, while fewer IBs were observed in the late m*Htt*-lowering group, compared with the LacQ140 mice (Supplemental Figure 6).

### mHtt lowering delayed transcriptional dysregulation in the striatum and cortex.

To assess the effect of m*Htt* lowering on the progression of LacQ140 transcriptional dysregulation, we performed RNA-Seq on the striatum and cortex isolated at 6 and 12 months of age. First, we compared the striatal and cortical transcriptomes from WT mice ± IPTG at 6 and 12 months of age to examine whether continuous IPTG treatment alone could influence gene expression. IPTG treatment affected the expression of 253 genes in the striatum and 356 genes in the cortex at 6 months of age, with 58 genes shared by the 2 tissues. At 12 months of age, 191 genes in the striatum and 17 genes in the cortex were affected, with no shared genes (Supplemental Table 2). To control for the effect of IPTG on the cortical and striatal transcriptomes, these genes were excluded from all subsequent analyses.

We next compared genes that were differentially expressed in the LacQ140 and WT ± IPTG striatal and cortical transcriptomes at 6 or 12 months of age using an FDR adjusted *P* < 0.05 and a fold change of at least 20% in either direction to identify LacQ140 transcriptionally dysregulated genes (Supplemental Figure 7 and Supplemental Table 3). In the striatum, we found 2,711 genes that were transcriptionally dysregulated at 6 months and 2,989 genes at 12 months, with 1,192 genes affected at both ages, defining a LacQ140 HD striatal signature of 4,508 dysregulated genes. In the cortex, 650 genes at 6 months and 164 genes at 12 months were dysregulated, with 25 genes shared at both ages (Supplemental Figure 8A and Supplemental Table 3).

**Figure 4:**
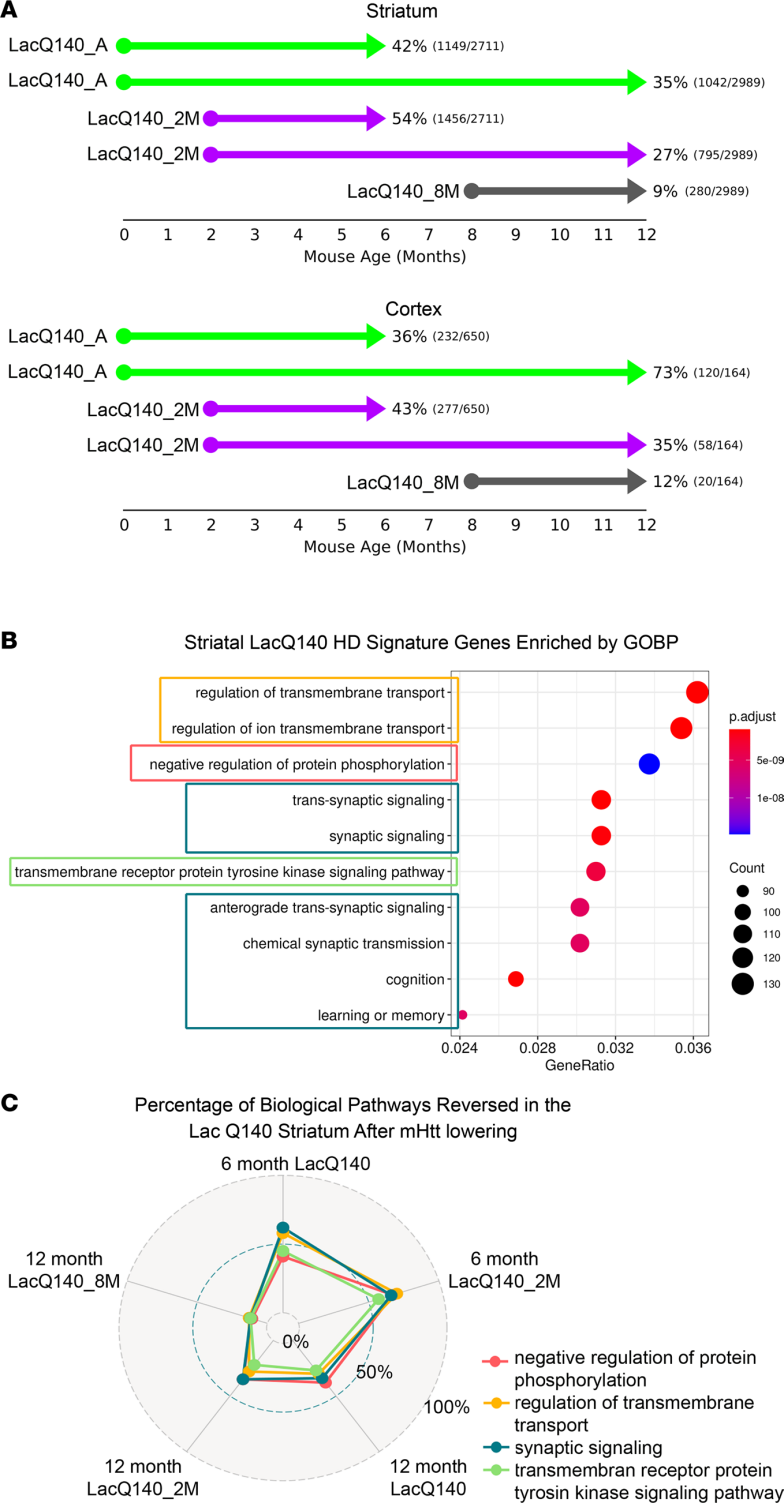
Transcriptional dysregulation is delayed with early but not late m*Htt* lowering. (**A**) After PP analysis, the number and percentage of LacQ140 HD signature genes that pass statistical significance for reversal are tallied in the striatum and cortex. Arrows indicate the duration of m*Htt* lowering; arrowheads represent age at tissue collection. Reversal is shown both as a percentage and as the number of reversed genes divided by the total number of LacQ140 HD signature genes. (**B**) In total, 4,508 genes in the 6- and 12-month LacQ140 HD model signature were analyzed for gene set overrepresentation against the GOBP collection to identify biological pathways that are dysregulated in the striatum of the model. Of the 64 gene sets that had *q* < 0.05, the top 10 ranked by the ratio of the number of HD signature genes in the gene set to the number represented in GOBP (gene ratio) are represented. Gene ratios represent the number of dysregulated genes that are represented in GOBP. Gene sets of related function are outlined; in cases such as those related to transmembrane transport (orange) or synaptic functions (teal), these gene sets largely overlap and can be represented by the member of the group that contains the most genes. (**C**) For each of the m*Htt*-lowering scenarios, striatal LacQ140 HD model signature genes within representative gene sets were tallied for their reversal percentages. These reversal results are represented as a radar plot. *n* = 5 males, *n* = 5 females/group, except 12-month striatum LacQ140 *n* = 5 males, *n* = 4 females.

To determine the probability that m*Htt* lowering attenuates LacQ140 transcriptional dysregulation on a gene-by-gene basis, we developed a posterior probability-based (PP-based) method to assign a reversal probability (RP) for each gene under different m*Htt*-lowering scenarios. The RP represents the probability of preventing or reversing transcriptional dysregulation. Comparing the numbers of reversed genes (fully, partially, and super-reversed genes) in each m*Htt*-lowering scenario revealed that continuous m*Htt* lowering from conception attenuated transcriptional dysregulation in the striatum and cortex at 6 months of age. Although the effect in the striatum diminished at 12 months of age, there was an increase in the percentage of genes with increased RP in the cortex (Figure 4A). It is notable that there is minimal overlap in cortical dysregulated genes between 6 and 12 months of age (Supplemental Figure 8A), suggesting different pathway dysregulation at different stages; very early m*Htt* lowering may be sufficient to more robustly slow transcriptional dysregulation in the cortex. Lowering m*Htt* from 2 months of age also alleviated transcriptional dysregulation in both cortex and striatum at 6 months of age, but this benefit diminished in both brain regions by 12 months of age, indicating a delay but not a halt in the progression of transcriptional dysregulation. Late m*Htt* lowering resulted in minimal attenuation of transcriptional dysregulation in both the striatum and cortex at 12 months of age (Figure 4A and Supplemental Table 4).

Examination of the RPs for the 4,508 striatal LacQ140 HD signature (combination of 6 and 12 months dysregulated striatal genes) revealed that, in the early m*Htt*-lowering groups, 42%–54% of the striatal LacQ140 dysregulated genes had a greater-than 95% chance of reversal at 6 months of age; however, the number of genes with an RP > 0.95 was greatly diminished by 12 months of age (27%–35%; Figure 4A and Supplemental Figure 8B).

Previously, we established a 266 dysregulated striatal gene signature shared by multiple HD mouse models (40). These 266 genes were also dysregulated in our LacQ140 model. When considering this multiple HD mouse model 266 gene signature, early m*Htt* lowering diminished transcriptional dysregulation by 71%–74% at 6 months of age and 31%–38% at 12 months of age, while late m*Htt* lowering had no beneficial effect (<1%) on ameliorating HD-associated gene expression changes at 12 months of age (Supplemental Table 5).

Enrichment analysis of gene ontology biological processes (GOBP) revealed that the gene sets most significantly overrepresented in the striatal LacQ140 dysregulated genes included genes associated with transmembrane transport, protein phosphorylation, synapse function, and transmembrane receptor protein tyrosine kinase signaling (Figure 4B). For each of these gene sets, the percentage of dysregulated genes, with expression changes attenuated by each m*Htt*-lowering scenario, was determined and presented in a radar plot (Figure 4C). These results demonstrate that, in the striatum, early m*Htt* lowering significantly sustained these cellular processes at 6 months of age (41%–62% RP in LacQ140_A and 57%–70% RP in LacQ140_2M), but the beneficial effects diminished with age, and by 12 months of age, 27%–38% RP in LacQ140_A and 22%–35% RP in LacQ140_2M was observed. In contrast, minimal attenuation (11%–13%) was achieved with late m*Htt* lowering when examined at 12 months of age.

**Figure 5:**
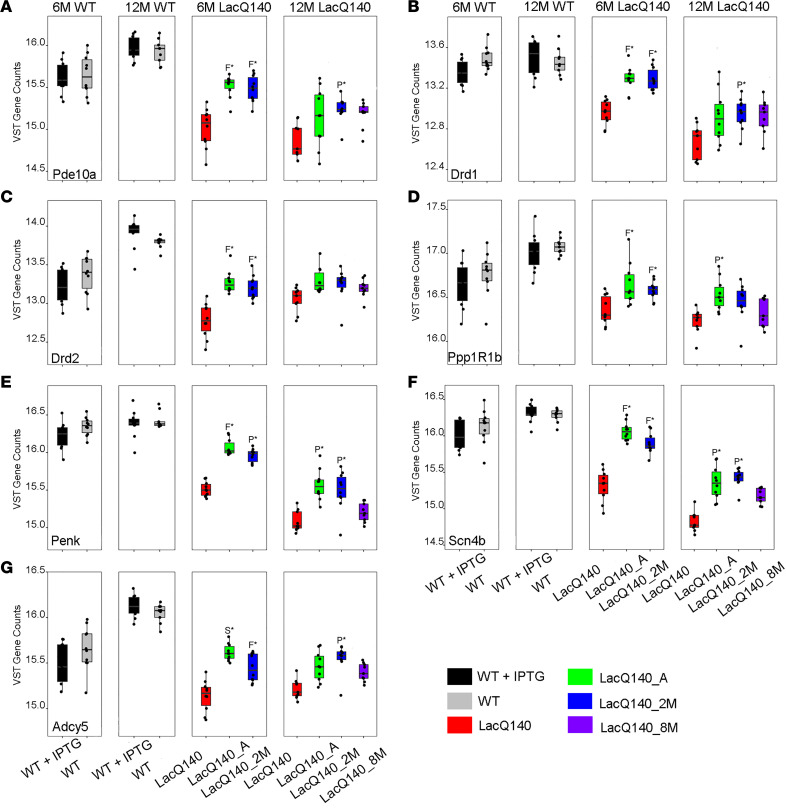
Transcriptional dysregulation of selected genes at 6 and 12 months of age is attenuated with m*Htt* lowering. (**A**–**G**) Expression levels for *Pde10a*, *Drd1*, *Drd2*, *Ppp1r1b*, *Adcy5*, *Penk*, and *Scn4b* in striatum are plotted as variance stabilizing transformed counts derived using DESeq2. For each m*Htt*-lowering scenario, the given gene’s RP is provided (full [F], partial [P], or super [S]). Asterisks represent overall reversal probability > 0.95; *n* = 5 males, *n* = 5 females/group, except 12-month striatum LacQ140 at *n* = 5 males, *n* = 4 females. Data are shown as mean ± SEM.

An examination of specific examples of commonly dysregulated synaptic genes in HD revealed that early m*Htt* lowering had a significant probability of fully preserving *Pde10a*, *Drd1*, *Scn4b*, *Adcy5*, *Drd2*, and *Ppp1r1b* expression levels at 6 months of age, but this beneficial effect was diminished by 12 months of age. Late m*Htt* lowering had no effect on attenuating the dysregulation of these genes (Figure 5).

### Early mHtt lowering delayed elevation of a neurodegeneration biomarker, neurofilament light chain.

**Figure 6:**
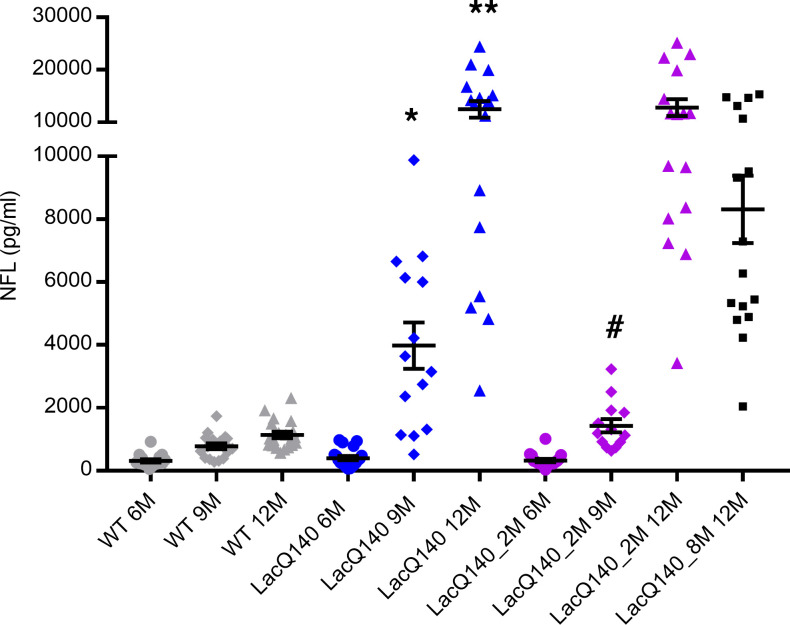
CSF neurofilament light chain (NFL) as a LacQ140 biomarker. WT mice received IPTG always (gray); LacQ140 mice received IPTG always (blue) for 2 (purple) or 8 (black) months of age and were sacrificed at 6, 9, or 12 months of age, and NFL was measured in CSF. NFL levels significantly increased in the LacQ140 CSF, compared with WT, starting at 9 months of age (1-way ANOVA), followed by Tukey’s multiple-comparison test. *P < 0.05, **P < 0.0001. There was a statistical difference between LacQ140 and LacQ140_2M at 9 months of age (unpaired *t* test); ^#^*P* < 0.005. LacQ140 (*n* = 19, 14, and 16 at 6, 9, and 12 months, respectively), LacQ140_2M (*n* = 18, 13, and 16 at 6, 9, and 12 months, respectively), and LacQ140_8M (*n* = 16), and WT (*n* = 17, 17, and 20 at 6, 9, and 12 months, respectively). Data are shown as mean ± SEM.

In patients with HD, an increase in NFL levels in the plasma and CSF have been reported to correlate with the onset of clinical symptoms and to increase during disease progression (41–45). Previously, NFL was reported to increase in the CSF of the R6/2 mouse model (46); therefore, we examined NFL levels in the CSF from mice with and without m*Htt* lowering. We observed a significant increase in CSF NFL levels from the LacQ140 HD model at 9 months of age, compared with WT. Early m*Htt* lowering at 2 months of age resulted in significantly lower NFL levels, compared with the LacQ140 mice at 9 months of age; however, neither early nor late global m*Htt* lowering prevented an increase in NFL levels in the CSF by 12 months age (Figure 6).

## Discussion

Many *HTT*-lowering therapeutics have entered or are now approaching clinical trials, but there are outstanding questions that need to be resolved. We generated and characterized an inducible mouse model, LacQ140, that can be used to examine the effects of global, allele-specific m*Htt* lowering at different times throughout the lifespan of the animals. This system responds well to orally administered IPTG, enabling the tuning of m*Htt* expression up or down in an experimentally simple manner; suppression is widespread, enabling reversible m*Htt*-specific lowering studies that cannot be achieved with any other current approach. Binding of *Lac* repressor to the *Lac* operator results in 27%–47% mHTT protein lowering throughout the brain, specifically 46%–47% lowering in the striatum and cortex, and 21%–68% mHTT lowering throughout the periphery. In addition, the expression of both full-length and *Htt1a* transcript can be regulated at the same time. *Htt*-lowering studies, including this one, usually rely on bulk dissection and quantification of m*Htt* in each tissue, meaning that the results are an average from cells with differing degrees of m*Htt* lowering. m*Htt* lowering in this model is likely to be more homogenous due to the ubiquitous β-actin promoter that we used to drive LacIR expression; however, further studies using RNA or BaseScope technologies, as well as single-cell sequencing (47, 48), to track m*Htt* expression at the cellular level will provide more information. Our LacQ140 mouse model exhibited progressive accumulation of mHTT protein aggregates, transcriptional dysregulation, and high content behavioral dysfunction similar to that reported for the Q140 mouse model (34, 49, 50). In addition, our LacQ140 model exhibited an age-dependent increase in NFL in the CSF, which is consistent with reports in HD carriers where there is an increase in NFL in the CSF (41, 42, 44). In this study, the expression of m*Htt* was lowered throughout the entire body, starting from conception (LacQ140_A), at 2 months of age (LacQ140_2M), or at 8 months of age (LacQ140_8M). The earlier m*Htt-*lowering groups allowed us to follow the mice long-term, for 10–12 months after m*Htt* lowering. Early m*Htt* lowering substantially delayed behavioral dysfunction, mHTT protein aggregation, transcriptional dysregulation, and elevation of CSF NFL levels; however, these benefits attenuated over time.

Although biochemical mHTT MSD analysis demonstrated a significant reduction in both soluble and aggregated mHTT, in all brain regions and ages examined after early or late m*Htt* lowering, the degree of suppression of aggregated mHTT, compared with LacQ140, declined with time and age at m*Htt* lowering. IHC detection of mHTT aggregation demonstrated a similar pattern; there was a substantial reduction in nuclear and extranuclear mHTT IBs with early m*Htt* lowering, especially at 6 months of age. In the striatum, late m*Htt* lowering had no effect on nuclear IBs measured at 12 months of age. Accumulation of aggregated mHTT in the nucleus, whether diffuse or in IBs, results in transcriptional dysregulation (51, 52), and the significant delay in mHTT aggregation observed here with early m*Htt* lowering likely explains the significant delay in transcriptional dysregulation. Despite this delay, mHTT aggregation was ongoing, and by 12 months, there was sufficient accumulation of aggregated mHTT in the nucleus to correlate with transcriptional dysregulation. In contrast, behavioral deficits have been correlated with cytoplasmic and extranuclear aggregates (51–53). Since the entire life cycle of mHTT aggregation was delayed in our m*Htt*-lowered groups, including the formation of extranuclear IBs, the window to delay onset of behavioral dysfunction may be longer. Behavioral improvement was observed 1 month after late m*Htt* lowering, although this result was confounded by the fact that this group of mice did not go through the behavioral testing with the other groups of mice when they were 6 months old. For this group, there may be some behaviors influenced by the novelty of the cubes at the 9-month time point only. Nonetheless, in all m*Htt*-lowered groups, there was an attenuation in behavioral benefits over time, with a significant reduction in behavior measured at 12 months, compared with 9 months in the very early and late m*Htt*-lowering groups. Since the end point of these experiments was 12 months of age, a longer follow-up, perhaps up to 18 months of age, may be needed to determine if m*Htt* early lowering’s benefits completely disappear long-term.

Although early m*Htt* lowering delayed an increase of NFL in the CSF, neither early nor late moderate global m*Htt* lowering could prevent or reverse the increase of NFL in the CSF when measured at 12 months of age. This is consistent with CSF NFL correlating with disease onset but not tracking with disease progression (44).

The global m*Htt* lowering exhibited in our model will be useful in the preclinical phase for discovering biomarkers that can be used to assess the distribution of m*Htt* lowering; for example, PET imaging with [^11^C]-CHDI-180R to track mHTT aggregates (54) revealed a significant reduction in mHTT aggregates in this model, when examined at 13 months of age, after either early or late m*Htt* lowering (55). Since m*Htt* levels are titratable, our LacQ140 HD mouse model could also be useful to study the benefits of a “Huntingtin holiday,” a therapeutic concept that posits that intermittent mHTT lowering for short periods of time could be beneficial (56). Our study indicates that moderate early m*Htt* lowering was able to significantly ameliorate multiple HD mouse model phenotypes for a finite period, but because disease progression was delayed and not halted, benefits diminished over time. Early m*Htt* lowering was also more beneficial than late m*Htt* lowering.

## Methods

Supplemental Methods are available online with this article.

### Mice.

The *LacO*/*LacIR*-regulatable mutant *Htt* allele was generated using gene targeting by inserting synthetic *LacO* sequences (31) flanking the transcriptional start site of *Htt^Q140^* (49) that expresses a full-length chimeric mouse/human m*Htt* with human exon 1 sequence encoding N terminal sequence, including an expanded polyQ stretch and the adjacent human proline-rich region. To introduce *LacO* sequences into the *Htt* promoter, 2 partially complementary oligonucleotides with an *Aat*II restriction site at the 5′ end of one oligonucleotide, 2 *LacO* sequences flanking the *Htt* transcriptional start site, and an *AlwN*I restriction site at the 5′end of the second oligonucleotide were annealed, repaired with the Klenow fragment of DNA polymerase I, and then digested with *AatI*I and *AlwN*I restriction enzymes to generate a *LacO*-modified DNA fragment corresponding to the WT *AatI*I-*AlwN*I restriction fragment spanning the 5′ end of the *Htt* exon 1. Oligonucleotides used were, HdhlacOPr forward: 5′-CATGACGTCACATTGTGAGCGCTCACAATGGGACGCACTGCCGCGA-3′, and HdhlacOPr reverse: 5′GGACAGACCCTGAAGACTTGGAGCCTACTGGCACTACGCGGCGCCACTTATTGTGAGCGCTCACAATAGCAGCAAGGCAATGAATGG-3′. To assemble the LacQ140 gene targeting vector, a synthetic *Aat*II-*AlwN*I DNA fragment containing the *LacO* modifications was used to replace the corresponding *AatI*I-*AlwN*I fragment in our CAG140 targeting vector (49). In addition, a neomycin phosphotransferase gene for positive selection driven by the phosphoglycerol kinase gene promoter (*pgk-neo*) flanked by *loxP* sites was inserted ~1.3 kb upstream of the *Htt* transcription initiation site. Transfected W9.5 ES clones were screened by Southern analysis, and mice were generated from the targeted ES cell clones using standard procedures. Germline transmission was obtained from 2 independently targeted ES cell clones, and mice were then backcrossed into the C57BL/6J genetic background using speed congenics. To control expression of *Htt*, *Htt^LacQ140^*mice were crossed with a transgenic line ubiquitously expressing the *E*. *coli* *Lac* repressor gene LacIR (31) (a gift from Heidi Scrable, University of Virginia, Charlottesville, Virginia, USA), also congenic in the C57BL/6J genetic background, to generate LacQ140 progeny. Genotype and CAG repeat number were confirmed by PCR sequencing of tail biopsies (Laragen Inc.). Mice had a sequenced CAG repeat length of 155 ± 3.3 (mean ± SD).

Mice were housed (uniform for sex, genotype, treatment) with enrichment (play tunnels, plastic bones, envirodry, and Bed-o’cobs) and fed ad libitum. To regulate m*Htt*, the lactose analog IPTG was added to the drinking water (at 10 mM, changed every 3–4 days) or chow (2.5 mg/g) to derepress the *LacQ140* allele and maintain maximal m*Htt* expression. To maintain normal m*Htt* expression levels during embryonic development, IPTG was administered to pregnant dams beginning at E5. IPTG was administered never or always, or it was withdrawn at 2 or 8 months. Mice used for behavioral testing were switched to IPTG-chow 3 days prior to PhenoCube testing and received IPTG chow (Envigo, AIN-93G) until they completed PhenoCube testing. IPTG-chow was stored at 4°C in the dark until delivered to mice. Fresh IPTG-chow was formulated for each time point that PhenoCube was run.

### RNA extraction and qPCR for quantification of Htt transcripts.

RNA was extracted using the RNeasy 96 Universal Tissue Kit (Qiagen) and quantified using a NanoDrop 8000 spectrophotometer (Thermo Fisher Scientific) and reverse transcribed into cDNA with random hexamers (Roche Applied Science). TaqMan Probes were used for Knock-in Htt (exon 1 to exon 2) (57); forward, 5′-CCTCCTCAGCTTCCTCAGC-3′; reverse, 5′-TGGTGGCTGAGAGTTCCTTC-3′. Housekeeping genes were *Atp5b*, *Canx* and *Rpl13a* for cortex and *Atp5b*, *Eif4a2* and *Gapdh* for striatum. Three independent RT reactions were performed for each RNA sample.

### Behavioral analysis.

Mice were tested in NeuroCube, SmartCube, and then PhenoCube (34–37) at 6, 9, and 12 months of age; LacQ140_8M underwent behavioral testing at 9 and 12 months of age.

### Analysis of high-content behavioral data.

To quantify the similarities and differences between groups, we conducted an analysis, using all collected behavioral features, termed DRFA. DRFA yielded a Discrimination Index that quantified the degree of overlap between the behavioral feature distributions of 2 experimental groups. To avoid overfitting and overinterpretation of certain features due to high correlation among them, we generated statistically independent combinations of the original features (decorrelated features). Each decorrelated feature was a statistically independent, weighted combination of all features. This achieves dimensionality reduction without loss of relevant information, which is essential for visualization and data interpretation. We used DRFA to generate Gaussian distributions approximating the groups of subjects in each given cohort (“clouds”) and estimated a quantitative measure of separability by calculating the overlap between the underlying probability distributions of the groups. Discrimination Indices were rescaled between 50% and 100%, where 50% represents no separation between 2 groups and 100% represents complete segregation.

We applied this method on the combined SmartCube, NeuroCube, and PhenoCube features data, grouping the samples as follows: LacQ140, 1 for each age; WTs groups consist of the 3 combined WT ± IPTG, 1 for each age; and 3 sets of m*Htt*-lowered groups, consisting of the LacQ140_A, LacQ140_2M, and LacQ140_8M, 1 for each age.

We first calculated the overall discrimination between the WTs and LacQ140 groups by testing the 6 groups within the same DRF. This 6-group DRF space captured differences due to age and to phenotype and its progression and, therefore, can be interpreted as providing “aging” and “disease” orthogonal axes. In this 6-group space, we also quantified the separation between specific pairs of m*Htt*-lowered groups and WT groups at each age, using the percent overlap between the corresponding distributions.

The 6-group space allowed for testing the effect of the m*Htt*-lowering manipulation in terms of disease and age axes, by projecting in the m*Htt*-lowered groups into this space. If the m*Htt* lowering completely and specifically rescues the disease phenotype of a group at a given age, we would expect maximal distance to the corresponding LacQ140 group (solid lines) and minimal distance to the corresponding WTs group (dotted lines; Figure 2). Any unspecific effect of the manipulation would result in a sideway orthogonal displacement with respect to the line connecting the LacQ140 and WTs groups. The significance level of the distance measured between the LacQ140 and m*Htt*-lowered groups were assessed with a *P* value based on the groups’ distributions.

### HTT protein quantitation using the Meso scale discovery (MSD) platform.

HTT quantitation with MSD has been previously described (58–60). The following antibody combinations were used: 2B7 and MW1 for detection of mHTT, and 2B7 and 4C9 for detection of aggregated mHTT (HD Community Biorepository at the Coriell Institute for Medical Research). Tissue homogenates were tested in technical triplicates.

### IHC and image acquisition.

Mice were perfused, brains were prepared, and IHC was performed as previously described (12). Primary antibodies included the following: monoclonal mouse anti-mHtt (1:3,000; PHP2, HD Community Biorepository at the Coriell Institute for Medical Research; ref. 39) and polyclonal guinea pig anti-NEUN (1/1000; MilliporeSigma, ABN90P). An antigen retrieval step was carried out for 30 minutes at 80°C in citrate buffer (0.01M Na-citrate buffer, pH 6.0). Secondary antibodies include the following: anti–mouse IgG (1/1,000, HRP-conjugated, Abcam, ab205719) and anti–guinea pig IgG (H + L) (1/1,000, CF-647, Sigma-Aldrich, SAB4600180). PHP2 signal was amplified using Biotinyl-Tyramide (TSA kit, Perkin Elmer, NEL700A001, 1:100 in 0.003% H_2_O_2_/0.1 M Borate Buffer, pH 8.5) and with streptavidin conjugated with Alexa-Fluor 488 (1:500 in 0.1% Triton/TBS; Vector Labs, SA-5488). Sections were mounted using aqueous mounting medium containing DAPI (Fluoroshield, MilliporeSigma, F6057) in 12-well glass-bottom plates (Sensoplate, Greiner, 665180) suitable for imaging with the Opera High Content Screening system (PerkinElmer Inc.).

Automated image acquisition was conducted using the Opera High Content Screening system and Opera software v.2.0.1 (PerkinElmer Inc.) using a ×40/1.15 numerical aperture water immersion objective (Olympus, pixel size 0.32 μm) for imaging of mHTT inclusions, as previously described (61). Image analysis scripts for characterization and quantification of mHTT inclusions were developed using Acapella Studio v.5.1 (PerkinElmer Inc.) and the integrated Acapella batch analysis as part of the Columbus system. Individual cells within tissue sections were identified using the DAPI signal and a general nuclei detection script based on the Acapella “nuclei detection B” algorithm. Specifically, the algorithm was defined to exclude nuclei with an area smaller than 200 px. Before detection of nuclei, noise and background signal was removed from the images by applying a sliding parabola filter (Acapella setting: curvature 6) on the DAPI signal.

Neurons were identified based on NeuN-ir signal intensity. The analysis of mHTT inclusions was performed based on the PHP2-ir signal intensity. First, a texture image was calculated using the SER Spot Texture Filter of Acapella 5.1 at a scale of 3 px. Spots were initially segmented as objects with a texture signal above 0.2. Objects smaller than 5 px or with an intensity lower than 2 times that of the mean intensity of the unfiltered image were excluded from the analysis. Furthermore, the mean spot intensity had to be 1.3-fold higher compared with the local surrounding (4 px wide ring around each spot). PHP2-ir spot density per mm^2^ in the striatum was quantified.

Image data from 3 sections were averaged per animal, and 4–6 animals per treatment group were used for statistical evaluation.

### RNA-Seq.

RNA was converted into indexed cDNA libraries and sequenced on the Illumina NovaSeq 6000 system to generate 40M paired-end reads at 100 bp read length per sample.

Differential expression tests were performed in R using DESeq2 with independent filtering disabled (62). Genes were considered significant if they had adjusted *P* < 0.05 after multiple-test correction and if they had fold changes of at least 20% in either direction. The LacQ140 versus WT contrast was used as the disease signature in each tissue, and genes responding to IPTG alone in the WT groups were removed from the disease signature.

### Determination of RNA-Seq phenotype modulations using PPs.

Analysis of RNA-Seq data comparing LacQ140 with WT striatum at 6M or 12M defined “LacQ140 HD signature” gene lists describing m*Htt*-induced transcriptional dysregulation based on the criteria specified above (fold change of at least 20% in either direction and adjusted *P* < 0.05 after multiple-test correction). The PP-based method was used to quantify the probability that m*Htt* lowering (LacQ140_A, LacQ140_2M, or LacQ140_8M) attenuated, either partially or fully, the transcriptional dysregulation on a gene-by-gene basis. This was done by viewing the effect of m*Htt* as a multiple of the HD model signature (in log_2_) — i.e., Δ_treat_ = αΔ_disease_. If α < 0, then the disease effect is reversed by the treatment; if α > 0, the disease effect is exacerbated by treatment.

The PP method examines 5 possible reversal classes for α: super-reversal: α < –1.3; full reversal: –1.3 < α < –0.7; partial reversal: –0.7 < α < –0.3; negligible reversal: –0.3 < α < 0.3; and exacerbation: α > 0.3.

The PP method takes a Bayesian approach and estimates the probability that α falls into each of the 5 reversal regions. Specifically, the estimated log_2_ fold change and standard error, as returned by the DESeq2 package’s “DESeq” function, are taken as the mean ± SD of a normal distribution, and we treat these as 2 independent distributions (treatment and disease effects). The probability that a gene is fully reversed is then given by: 


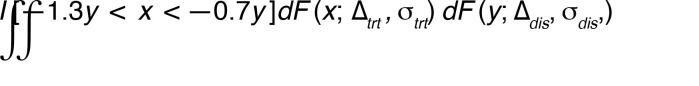


(and so on for the other classes), where *F(x;μ,σ)* is the normal density measure with mean μ and SD σ. The equation above was implemented using quasi-Monte Carlo integration. We use 0.83 percentiles (121 points) of the univariate marginal normal distributions arranged in a grid of 121 × 121 test points, with the bivariate normal density serving as importance weights.

Once the probabilities for each region were calculated, each gene was assigned to a reversal category by the following logic: If *P*(negligible reversible) ≥ 0.05, the gene is considered to be negligibly reversed and was assigned to the negligible reversal category. Otherwise, the gene was assigned to the reversal category with the highest probability. These calculations were performed for all the lowering scenarios at 6 and 12 months of age. The Overall RP (ORP) for each gene is also reported as 1 – *P*[negligible reversal]. An ORP > 0.95 is considered statistically significant.

### Gene set overrepresentation analysis of RNA-Seq LacQ140 phenotype modulations in the striatum.

The LacQ140 HD signature dysregulated gene lists for striatal samples at 6M and 12M were compiled based on gene expression changes between LacQ140 and WT in the striatum at both ages; an intersecting list of 1,192 genes was derived as the “HD intersect” gene list. This list of genes was tested for gene set overrepresentation against the GOBP version 18.9.29 using the enricher function within the R clusterProfiler package (63, 64). Gene sets with a clusterProfiler *q* < 0.05 were deemed significant. The HD signature genes within each gene set were then scored for normalization based on its ORP. In this way, the percentage of HD signature genes present in each gene set was determined.

### CSF collection and NFL quantification.

Animals were anesthetized with isoflurane and placed onto a stereotaxic frame. The back of the animal’s neck was shaved, and an incision was made to expose the cisterna magna. A pulled glass capillary pipette was inserted about 2 mm into the cisternal space, and CSF was collected. CSF samples were assayed at 1:4,000 dilution according to the manufacturer’s recommendations (Quanterix, 103400). Sample values (AEB units) were measured, and NFL protein concentrations (pg/mL) were interpolated from a calibration curve.

### Data availability.

RNA-Seq data that support the findings of this study have been deposited in GEO with the accession no. GSE156236.

### Statistics.

All values are presented as mean ± SEM, and differences were considered statistically significant at the *P* < 0.05 level. Statistical analyses were performed using GraphPad Prism 8 and R software. For comparisons between 2 groups, unpaired and paired 2-tailed *t* tests were used. Differences between all group means were analyzed by 1-way ANOVA to test for the difference between the groups. For post hoc tests, Bonferroni’s multiple comparison tests were used to compare means to a selected mean; Tukey’s test was used to compare all means against each other; Dunnett’s multiple-comparison tests were used to compare means with a control mean. When assumptions for parametric tests were not met, nonparametric data were analyzed with a Kruskal-Wallis test, followed by Dunn’s multiple-comparison test. Statistical details for transcriptional and behavioral data are described in detail above.

For high-content behavioral analysis, *P* values for the discrimination indices are estimated by nonparametric statistical analysis, based on 1,000 random permutations of the sample group labels. In visualizations of the cloud analysis, clouds are centered around the mean values, with an inner circle indicating the SEM and outside circle indicating the SD (35). The effects of each m*Htt-*lowering treatment at the different test ages were analyzed using the calculated discrimination indices betweenLacQ140 and m*Htt-*lowered groups at each age. Discriminations between the lowered groups (green clouds; Figure 2) at each age are marked with continuous lines joining the LacQ140 groups. A first step was conducted by projecting the m*Htt*-lowered individual data orthogonally onto the axis joining the corresponding LacQ140 and WTs groups. The individuals’ projected data points were then normalized between 0 (overlap with LacQ140) and 100 (overlap with WTs). An analysis was done to evaluate the effects of age (2 or 3 testing ages) and treatment (3 different m*Htt*-lowering regimens) as factors and their interaction. Data were evaluated via 2-way repeated-measured ANOVA carried out with SAS (SAS Institute Inc.) using mixed-effect models, based on restricted maximum likelihood estimation. Random factors included age (for repeated measures) and a random intercept, to adjust for fluctuations between individual animals. The covariance structure for the repeated measures was autoregressive-1 and the random intercept used a variance component covariance structure. Degrees of freedom were adjusted based on the Kenward-Roger method. An effect was considered significant if *P* < 0.05. Significant interactions were followed up with simple main effects analyses (slice effects analysis). For simple main effects analyses, *P* values were adjusted via simulation. An effect was considered significant if the adjusted *P* < 0.05.

### Study approval.

All animal experiments used humane end points, were performed according to the *Guide for the Care and Use of Laboratory Animals* (National Academies Press, 2011)**,** and were approved by the University of Virginia Animal Care and Use Committee (ACUC). PsychoGenics IACUC reviewed all procedures and behavioral test protocols.

## Author contributions

Conception and design of the studies were contributed by DMM, ES, IMS, DH, and SOZ. qPCR was contributed by JPL and MK. QuantiGene was contributed by LR. MSD was contributed by K Kuhlbrodt. IHC was contributed by KT and FP. In vivo work and Cube experiments were contributed by K Kerker. Statistics were contributed by MA. Supervision of the experiments was contributed by DMM, KC, VK, BL, JR, JA, ES, IMS, DH, and SOZ. Interpretation of results was contributed by DMM, JPL, AAI, MB, RM, DB, SR, JO, JRG, CH, VK, JR, JA, IMS, DH, and SOZ. Preparation of final figures was contributed by DMM and SOZ. Writing the original draft was contributed by DMM and SOZ.

## Supplementary Material

Supplemental data

Supplemental table 1

Supplemental table 2

Supplemental table 3

Supplemental table 4

Supplemental table 5

## Version 1 10/24/2022

Electronic publication
