# Supplementary material for: Benefits of global mutant huntingtin lowering diminish over time in a Huntington’s disease mouse model
Source: JCI Insight. 2022 Oct 24;7(20):e161769. doi: 10.1172/jci.insight.161769 (PMC9714791; doi:10.1172/jci.insight.161769)
Supplement: Supplemental data [file jciinsight-7-161769-s246.pdf]

Supplemental Methods

Pharmacokinetics, pharmacodynamics, and stability of IPTG

2-month-old LacQ140 mice were given 10mM IPTG in drinking water for 1 week and 4-month-old LacQ140 mice were given IPTG in chow (2.5 mg/g) for 4 weeks and then anesthetized; blood, and cortex from one brain hemisphere and the other brain hemisphere were flash frozen. The concentration of IPTG in plasma and brain were determined utilizing UPLC-MS/MS methods. Plasma and brain were extracted with acetonitrile (1:4; v:v). Extracts were dried and reconstituted with an internal standard (IS) solution (warfarin, 5 ng/mL) andinjected on a Waters Acquity column (1.8 µM, 2.1x50 mm) using a gradient of 100% mobile phase A (ammonium bicarbonate, pH 10) to 100% mobile phase B (acetonitrile) in 2 min with a flow rate of 0.6 mL/min. The eluant was monitored for IPTG (parent, m/z 237.1, product 161.0) and warfarin (parent m/z 307.2, product m/z 160.9) using Sciex API 5000 (Supplemental Figure 9A). To determine the pharmacodynamic effect of IPTG, qPCR was used to measure relative mRNA expression of cortical m*Htt* with or without IPTG (Supplemental Figure 9B).

7.5 mg/g IPTG was formulated in AIN-93G chow. To determine stability of IPTG in chow, the pellets were pulverized, extracted with water and acetonitrile, and then analyzed by a UPLC-MS/MS methods as stated above for plasma and brain (Supplemental Figure 9C).

***Htt1a* transcript quantification**

Purified cortical RNA was used to detect intronic variants of *Htt*, including *Htt1a*, using a custom-prepared bDNA QuantiGene Plex set and QuantiGene Plex Assay Kits as described previously (1). Target expression levels were normalized using the geometrical mean of the housekeeping genes *Atp5b* and *Canx.*

Kinetics of m*Htt* mRNA and protein reduction following IPTG withdrawal

To assess m*Htt* reduction following IPTG withdrawal, LacQ140 mice were treated with IPTG from E5 until 6 months of age. Cortex, striatum, hippocampus, cerebellum, liver, white and brown adipose tissues, pancreas, heart, and skeletal muscles were isolated from each mouse at 0, 3, 6, 10, 15, 20, and 25 days after IPTG withdrawal and snap frozen. Total RNA was purified from dissected tissues using an Aurum Total RNA Fatty and Fibrous Tissue kit (Biorad 7326830) and then quantified with a Nanodrop 1000 spectrophotometer and reverse transcribed using an iScript cDNA synthesis kit (Biorad 1708891) and used for each droplet digital PCR (ddPCR) reaction with the ddPCR supermix for probes (Biorad 1863023) using the cycling condition recommended by the manufacture. The primers used to amplify the 7Q-*Htt* (wild type *Htt*) cDNA are: 5'-ACCGCCGCTGCCAG-3' and 5'-TCTTTCTTGGTGGCTGAGAGT-3', and the probe used to recognize the 7Q-*Htt* PCR product is: HEX-5'-CGGCAGAGGAACCGCT-3'-Iowa Black FQ. The primers used to amplify the 140Q-*Htt* cDNA are: 5'-ACCCGGCCCGGCT-3' and 5'-TCTTTCTTGGTGGCTGAGAGT-3', and the probe used to recognize the 140Q-*Htt* PCR product is: FAM-5'-TGGCTGAGGAGCCGCT-3'-Iowa Black FQ.

To examine mHTT protein levels following IPTG withdrawal, tissues were homogenized in RIPA buffer (25mM Tris pH7.6, 150mM NaCl, 1% NP40, 1% Sodium deoxycholate, 0.01% SDS, 1mM EDTA, 1mM DTT) supplemented with Halt protease inhibitors (ThermoScientific PI 78425), and centrifuged at 15k for 10 min at 4˚C. Western blotting was performed as described in (2). Primary antibodies used were: MAB2166 (Millipore, 1:1000), mTOR (Cell Signaling 2972S, 1:1000), LacI (Rockland Immuno, 600-401-B04, 1:1000) and β-actin (Cell Signaling 3700, 1:1000). Images were captured with a LiCor Odyssey Fc imager and quantified with. Image Studio software (LiCor).

1. Papadopoulou AS, Gomez-Paredes C, Mason MA, Taxy BA, Howland D, and Bates GP. Extensive Expression Analysis of Htt Transcripts in Brain Regions from the zQ175 HD Mouse Model Using a QuantiGene Multiplex Assay. *Scientific reports.* 2019;9(1):16137.
2. Clabough EB, and Zeitlin SO. Deletion of the triplet repeat encoding polyglutamine within the mouse Huntington's disease gene results in subtle behavioral/motor phenotypes in vivo and elevated levels of ATP with cellular senescence in vitro. *Hum Mol Genet.* 2006;15(4):607-23.
3. John C. Obenauer JC, Viktoria Andreeva, Jeffrey S. Aaronson, Ramee Lee, Andrea Caricasole, Jim Rosinski. Expression analysis of Huntington disease mouse models reveals robust striatum disease signatures. *BioRxiv.* 2022;doi: 10.1101/2022.02.04.479180.

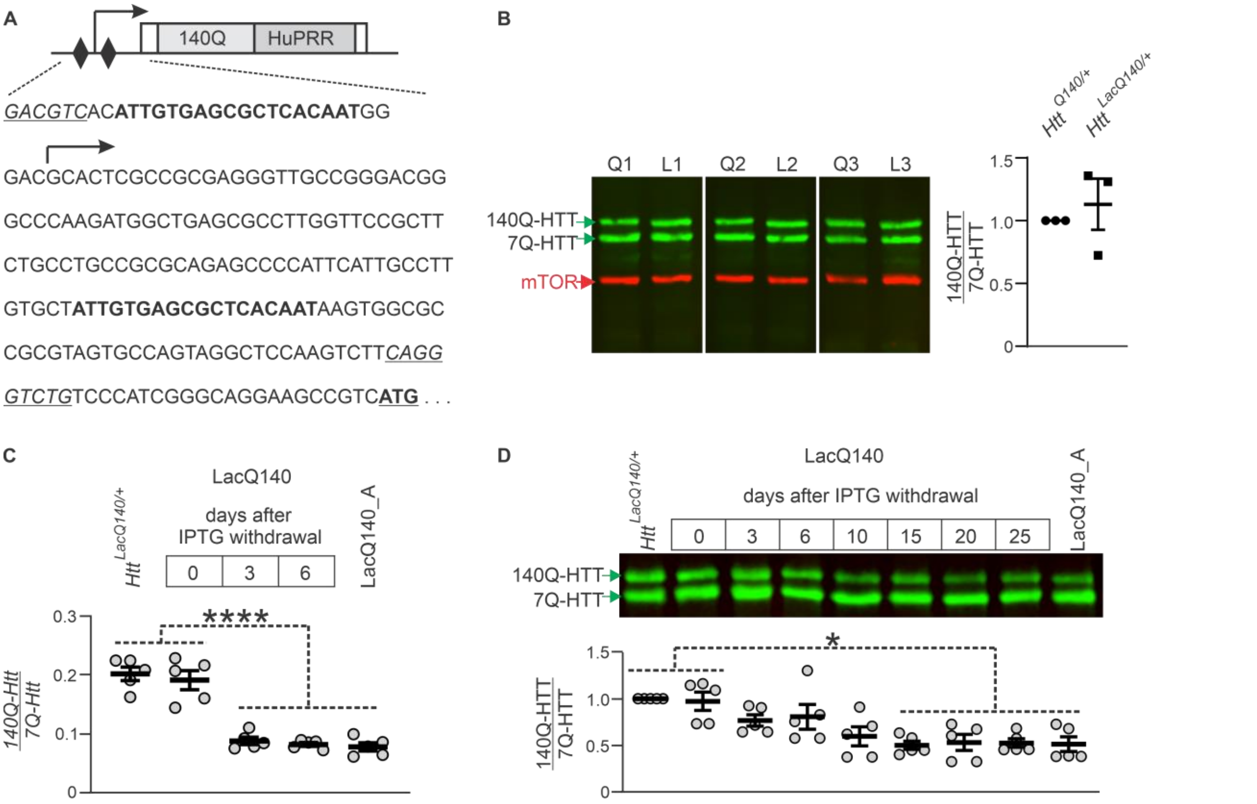

Supplemental Figure 1. Schematic of $Htt^{LacQ140}$ and time course of m*Htt* repression following IPTG withdrawal. (A) Schematic of a portion of the $Htt^{LacQ140}$ allele showing the proximal promoter region, exon 1, and a small portion of intron 1. Black diamonds represent the *Lac* operator sequences flanking the transcription start site (black arrow). The expanded CAG repeat encoding 140Q and the adjacent sequence encoding the human proline-rich region (HuPRR) are shown not to scale. The sequence of the LacQ140 promoter region showing the *Lac* operator elements (in bold), the Methionine translation initiation codon (bold and underlined), and *Aat*II and *Alw*N1 restriction sites (italic and underlined). (B) Western blots (MAB2166) and quantitation of 140Q-HTT levels relative to 7Q-HTT levels in whole brain protein isolated from 2-month-old $Htt^{Q140/+}$ (Q1-3) and $Htt^{LacQ140/+}$ (L1-3) mice. The positions of 140Q-HTT and 7Q-HTT are indicated (green arrows), along with mTOR as a loading control (red arrow). No significant differences in 140Q-HTT levels were observed in a comparison of the two groups, Paired t-test; (n=3/group, mean ± SEM). (C) LacQ140 mice were continuously provided with IPTG in their drinking water until they reached 6-months of age, then euthanized at 0, 3 or 6 days after IPTG withdrawal. RNA from dissected cortex was isolated and used to quantify the *140Q-Htt*/*7Q-Htt* cDNA ratio by RT-ddPCR in comparison to $Htt^{LacQ140}$ and LacQ`140_A controls. ****$p<0.001$, One-way ANOVA (n=5/group, mean ± SEM). (D) Western blot (MAB2166) and quantitation of 140Q-HTT levels relative to 7Q-HTT levels in cortical protein isolated from LacQ140 mice on the last day of IPTG treatment (Day 0), and days 3-25 following IPTG withdrawal. Cortical protein extracts from 6-month-old $Htt^{LacQ140}$ and LacQ140_A mice were used as controls. The positions of 140Q-HTT and 7Q-HTT are indicated (green arrows), *$p<0.05$, One-way ANOVA with Tukey's multiple comparison; (n=5/group (3 males and 2 females, mean ± SEM).

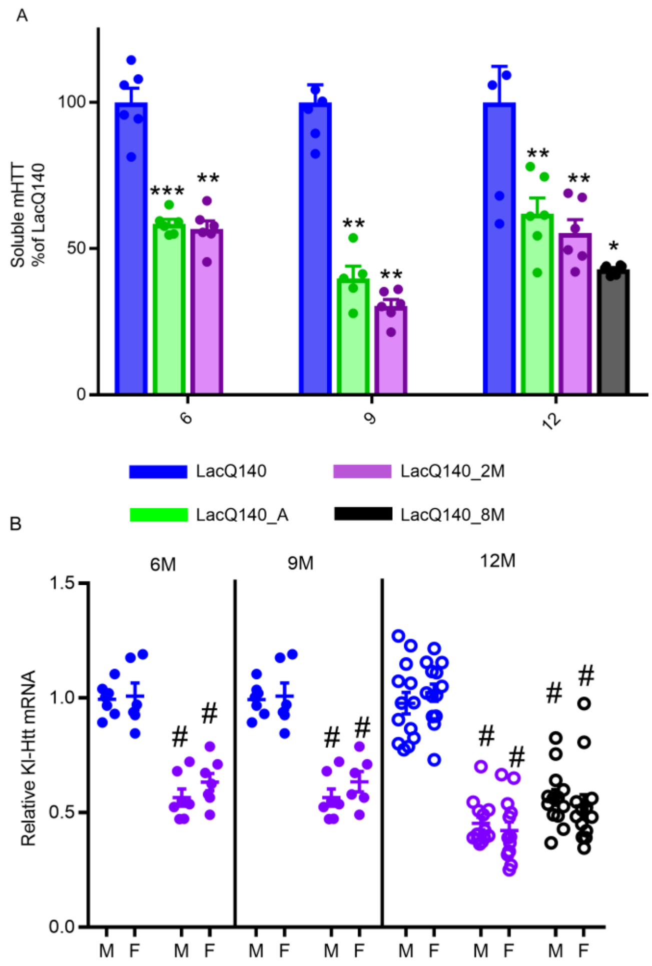

Supplemental Figure 2. The Operator-Repressor system regulated m*Htt* consistently across age and sex. LacQ140■, LacQ140_A ■, and LacQ140_2M■ were sacrificed at 6, 9 and 12 months of age, while LacQ140_8M ■ were euthanized at 12 months of age. (A) Soluble mHTT was measured using 2B7-MW1 MSD in the cerebellum. There were no age-dependent differences in mHTT levels in the LacQ140 at 6, 9 and 12 months (One-way ANOVA). Similarly, there was no age-dependent difference in mHTT levels between the LacQ140_A or LacQ140_2M at 6, 9 and 12 months (One-way ANOVA). At each age there was a significant reduction in the m*Htt*-lowered groups (LacQ140_A, LacQ140_2M and LacQ140_8M), compared to LacQ140, ***p < 0.0001, **p < 0.001, *p < 0.005 Two-tailed t-test, (n= 6/group, mean ± SEM); MSD signal in arbitrary units (AU). (B) Relative mRNA expression of m*Htt*, normalized to the geometric means of 3 housekeeping genes was measured using qPCR in the cerebellum. Data is represented as the average of three independent qPCR reactions. Each age was run separately, therefore LacQ140 at each age was set to 1.0. At each age, there was a significant reduction in the m*Htt* lowered groups (LacQ140_2M and LacQ140_8M), compared to LacQ140 (One-Way ANOVA, followed by Tukey's multiple comparisons test, # p < 0.0001). There were no sex-specific differences (One-Way ANOVA). LacQ140: n=8 males, n=7 females at 6M and 9M; n=13/sex at 12M; LacQ140_2M: n=8/sex at 6M; n=8 males, n=7 females at 9M; n=13/sex at 12M; LacQ140_8M: n=13/sex.

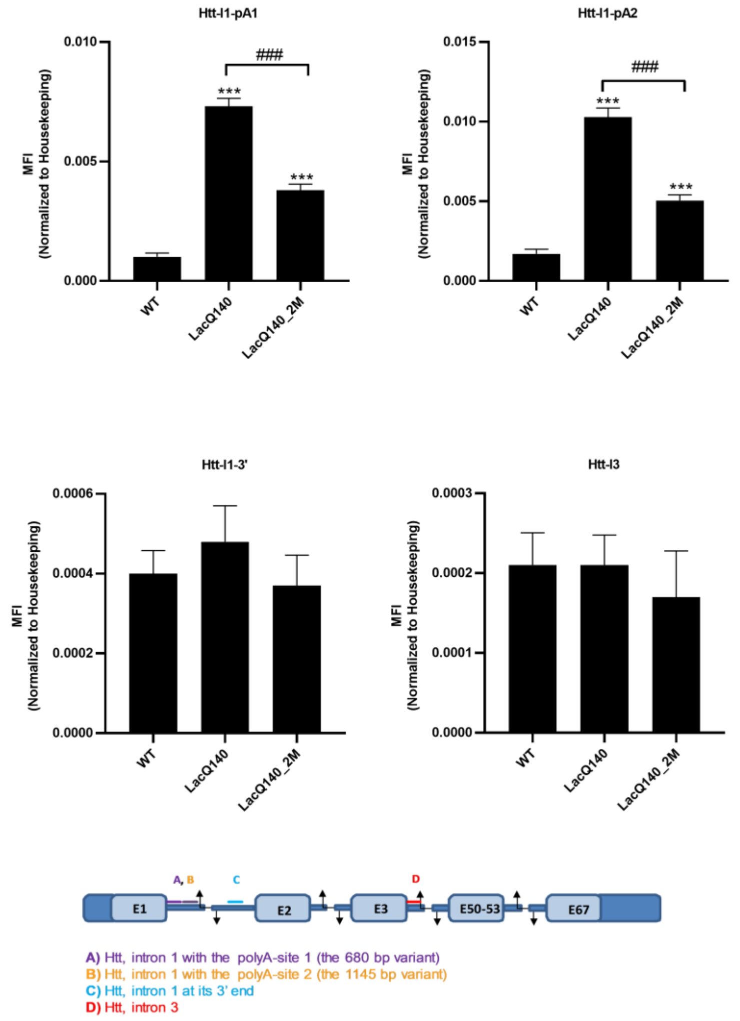

Supplemental Figure 3. *Htt1a* transcripts were lowered in the cortex of LacQ140 mice after IPTG removal. The expression levels of different intronic *Htt* transcripts in the cortex of 6-month-old WT + IPTG, LacQ140 and LacQ140_2M mice. Expression levels are presented as mean fluorescence intensity (MFI) normalized to the geometric mean of the housekeeping genes *Atp5b* and *Canx* (with ± SEM). The I_1-pA_1 probe set identifies the *Htt1a* transcripts that terminate at both the first and the second cryptic poly(A) signals. The I_1-pA_2 probe set recognizes only the *Htt1a* transcript that terminates at the second poly(A) signal. These transcripts were increased in the LacQ140 model, compared to WT. After m*Htt* lowering, *Htt1a* transcripts were significantly reduced. One-way ANOVA with Tukey's multiple comparison test, ***p < 0.001 (against the WT group) and $^{###}$p < 0.001 (pairwise comparisons indicated). The I_1-3′ probe set identifies incompletely spliced intron 1 sequences that have not terminated at cryptic poly(A) signals, while the I_3 probe set serves to control for any contaminating *Htt* pre-mRNA. There were no significant differences in the sequences identified by these 2 control probes, Kruskal-Wallis test. N=10 (5 males, 5 females) for each group.

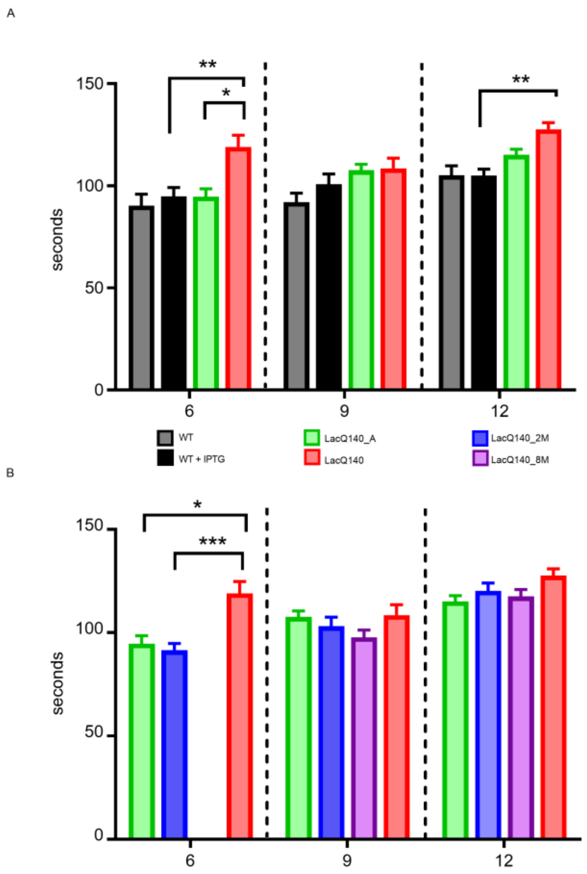

Supplemental Figure 4. Immobility in LacQ140 and m*Htt* lowered groups across age. (A) To determine if IPTG treatment effected the motor phenotype (measuring immobility) of the HD models, and if such effect interacted with age, we conducted a Mixed Model ANOVA, including Genotype as a factor. There was a significant effect of Genotype, no interaction of Genotype and Treatment but a significant interaction of Genotype, Treatment, and Age (F(1,104)=18.3, *p*<0.0001; F(1,104)=1.5, *p*=0.217; F(4,141)=3.4, *p*<0.01, respectively). There was a significant difference between the LacQ140 and its corresponding WT group (with IPTG treatment) at both 6 and 12 months of age (*p*s<0.01, Tukey-Kramer) but no difference between the LacQ140_A and its corresponding WT group (without IPTG). Interestingly, there was a statistically significant difference between LacQ140_A and LacQ140 groups at 6 months of age (*p*<0.01, Tukey-Kramer). (B) As the LacQ140 mice were significantly less mobile than their corresponding WT group, we asked whether lower m*Htt* at different ages would be associated with higher mobility. A Mixed Model ANOVA with Treatment (comprising different m*Htt* lowering regimens) and Age as factors found that both Treatment and its interaction with Age were significant (F(3,113)= 5.3, *p*<0.01 and F(5,139)= 3.9, *p*<0.01, respectively). Consistently with the previous analysis in (A), we observed a statistically significant difference between LacQ140_A and LacQ140 groups at 6 months of age (*p*<0.05, Tukey-Kramer). We also observed a statistically significant difference between LacQ140_2M and LacQ140 at 6 months of age (*p*<0.001, Tukey-Kramer). *p<0.05, **p<0.01, ***p<0.001

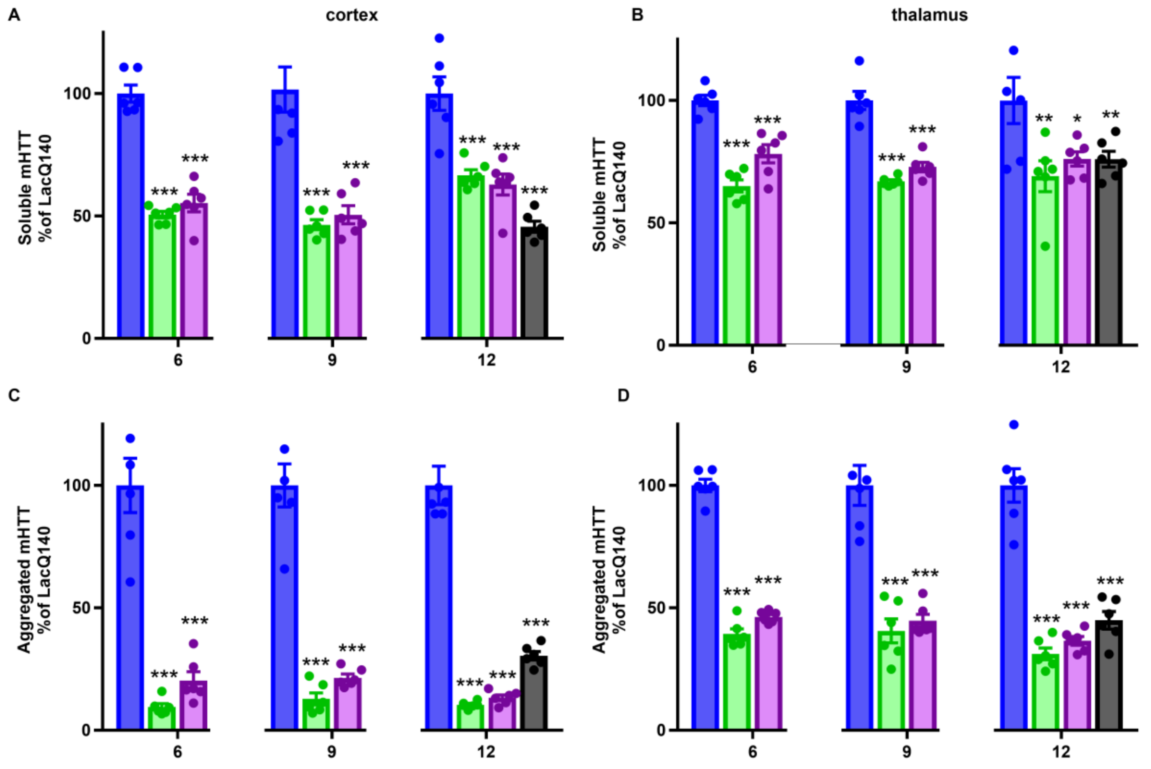

Supplemental Figure 5. mHTT protein lowering in cortex and thalamus. LacQ140■, LacQ140_A ■, and LacQ140_2M■ were sacrificed at 6, 9 and 12 months of age, while LacQ140_8M■ were sacrificed at 12 months of age. Data were normalized to LacQ140, set at 100% for each age. Soluble mHTT was measured using 2B7-MW1 MSD in the (A) cortex and (B) thalamus. One-way ANOVA, followed by Bonferroni's multiple comparison test *** $p \leq 0.0001$, ** $p < 0.01$, * $p < 0.02$ (n= 6/group). Aggregated mHTT was measured using MW8-4C9 MSD in the (C) cortex and (D) thalamus. One-way ANOVA, followed by Bonferroni's multiple comparison test *** $p < 0.0001$ (n= 6/group).

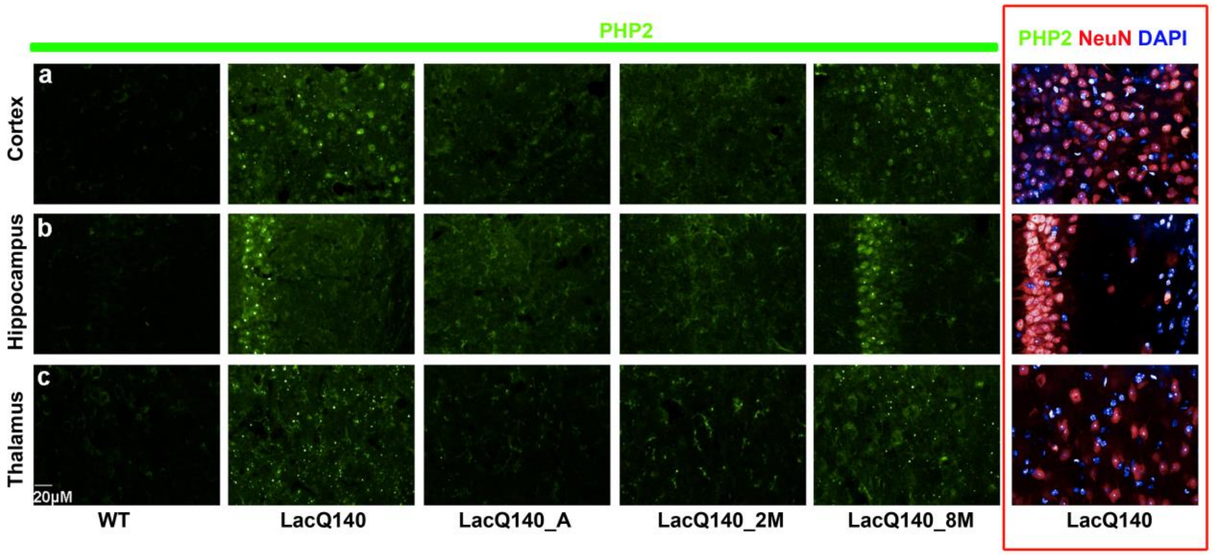

Supplemental Figure 6. mHTT aggregation in the cortex, hippocampus and thalamus. LacQ140, LacQ140_A, LacQ140_2M and LacQ140_8M mice were sacrificed at 12 months of age. Representative PHP2 immunolabeling of (a) cortex, (b) CA1 hippocampus and (c) thalamus; scale bar=20µm. mHTT (PHP2), green; neurons (NeuN), red; DAPI, blue. The same LacQ140 images were used in both the PHP2 only and PHP2/NeuN/DAPI images.

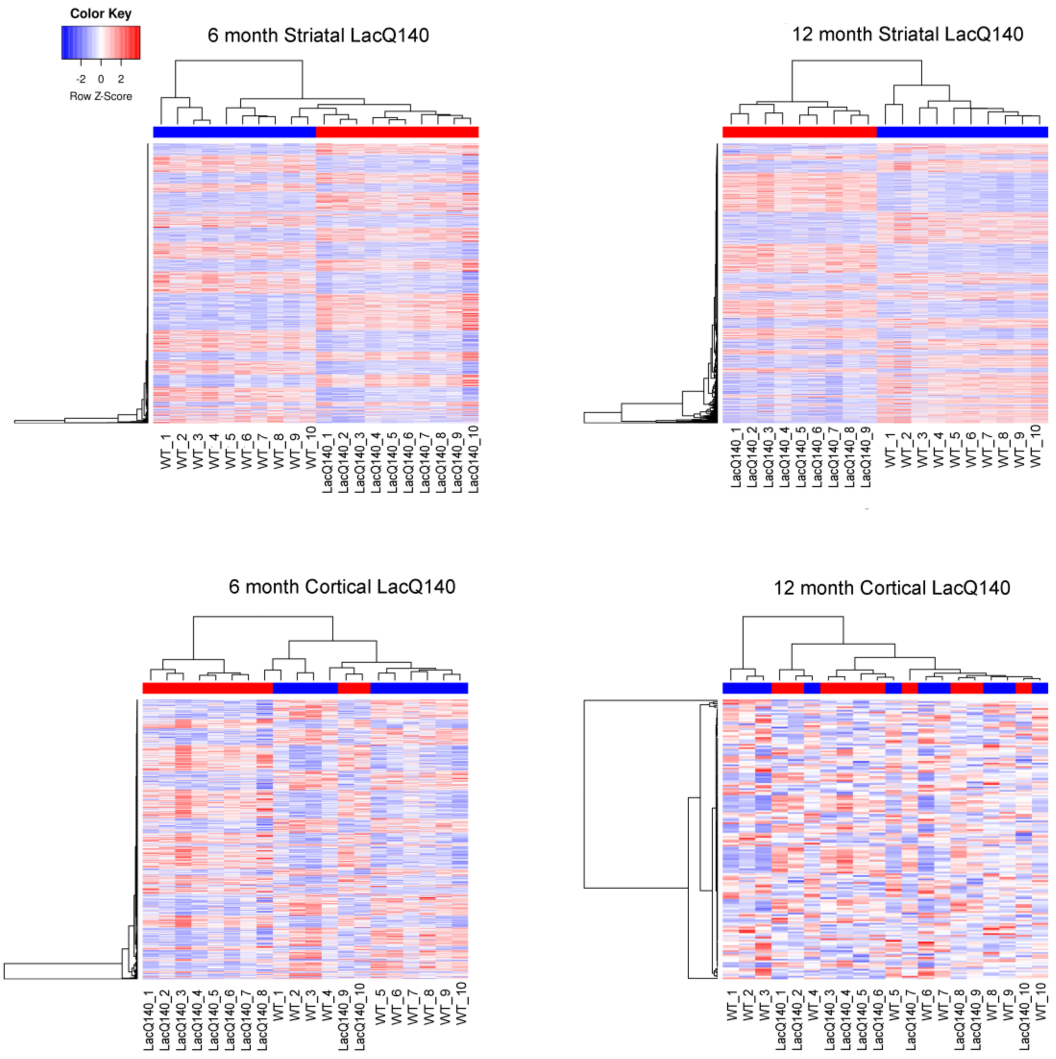

Supplemental Figure 7. Transcriptional dysregulation in the LacQ140 mouse model. Heatmaps are shown for significantly dysregulated genes in the striatum of LacQ140, compared to WT, at 6 months and 12 months; and in the cortex at 6 months and 12 months. Genes were considered significant if they had adjusted p-values < 0.05 after multiple test correction and if they had fold-changes of at least 20% in either direction. Red indicates higher expression and blue indicates lower expression, scaled as Z scores of the sample values in each row of the heatmap. At the top of each heatmap, red indicates disease samples and blue indicates wild type samples; hierarchical clustering of the cortex samples at 12 months shows some mixing of the LacQ140 and WT samples due to a relatively weak disease signature (n=5 males, n=5 females/group, except 12M striatum LacQ140 n=5 males, n=4 females).

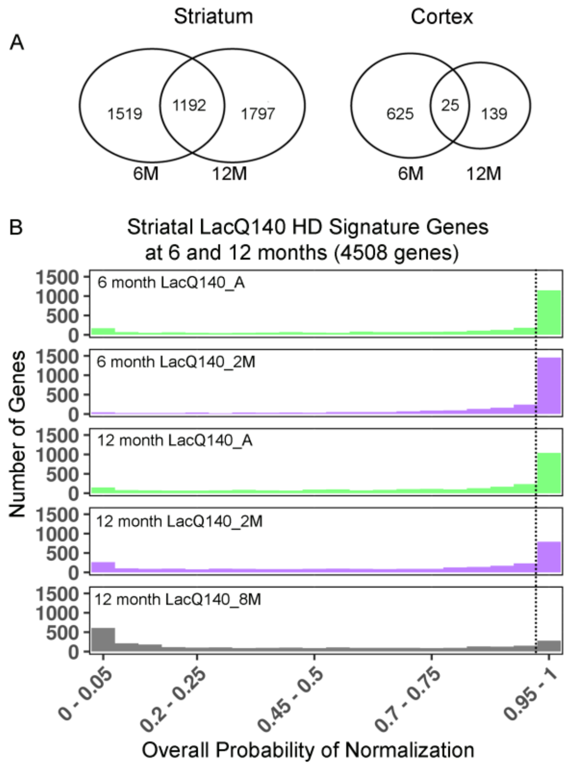

Supplemental Figure 8. Overlap in LacQ140 dysregulated across ages and distribution of genes probability of reversal. We identified a signature of dysregulated striatal genes in the LacQ140 model; the signature was defined by differential gene expression changes in WT versus LacQ140 using an FDR adjusted p-value < 0.05 and a fold change of $\geq 20\%$. 4508 genes were dysregulated at 6 and/or 12 months of age. 2711 genes were dysregulated at 6 months of age and 2989 genes were dysregulated at 12 months of age. There was an overlap of 1192 genes dysregulated at both 6 and 12 months, yielding 4508 dysregulated striatal genes in this model. A Venn diagram of gene overlap is presented in (A). All 4508 dysregulated genes were examined in each of the m*Htt* lowered groups and the probability of reversal of that dysregulation was calculated with a 0.95 cutoff for rescue probability. We plotted the distribution of rescue probabilities and observed the majority of genes were in the 0.95-1.0 or 0-0.5 category, to confirm that the 0.95 cutoff criteria was appropriate and there were not a large number of genes that just missed the cutoff (B).

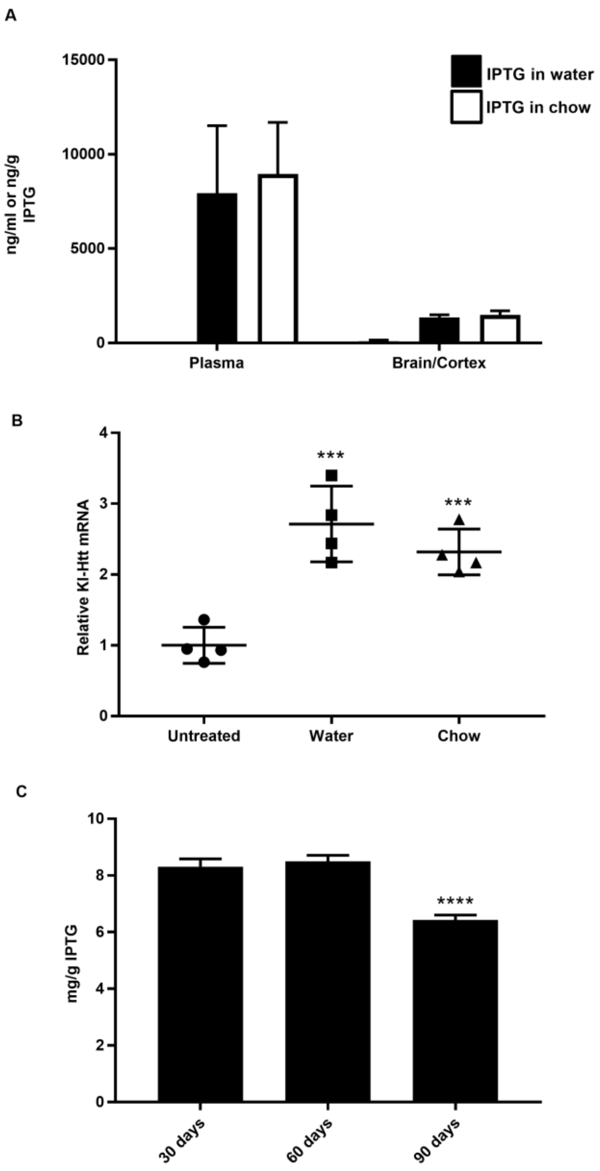

Supplemental Figure 9. IPTG PK/PD and stability. (A) After 1 week exposure of 10nM IPTG in drinking water in 2-month-old LacQ140 mice, IPTG was detected in plasma and hemibrains in treated groups (■ n=4, mean ± SEM). Similar levels of IPTG were detected in the plasma and cortex of 4-month-old LacQ140 mice after 28 days of 2.5mg/g IPTG in chow (□ n=4, mean ± SEM). (B) Relative mRNA expression of cortical m*Htt*, normalized to the geometric means of 3 housekeeping genes. Data are represented as average of three independent RT reactions. IPTG treatment resulted in greater expression of knock-in m*Htt* mRNA expression, compared to untreated *** $p < 0.001$, Unpaired t-test to compare IPTG-treated water or chow to untreated (n=4, mean ± SEM). (C) IPTG chow prepared at 7.5 mg/g was stable in chow for 60 days; **** $p < 0.0001$, ANOVA with Dunnett's multiple comparison (n=10 pellet samples/time).

Supplementary Table 1. Behavioral features. Features were collected from three high-throughput Psychogenics platforms (SmartCube®, NeuroCube® and PhenoCube®). Animal ID and group are presented with values for the top features measured in this study. Immobility_5 is highlighted in blue and was used for analysis in Supplemental Figure 4.

Supplementary Table 2. IPTG effects on gene expression. To identify gene expression changes influenced by IPTG, RNASeq was performed on the striatum and cortex of WT mice with and without IPTG at 6 and 12 months of age. Ensembl gene ID and gene names are presented with log fold change between WT with and without IPTG, the standard error, and adjusted p-value.

Supplementary Table 3. Differential gene expression. There are 4 tabs: 6M striatum, 12M striatum, 6M cortex, 12M cortex. Ensembl gene ID, gene names and gene descriptions, raw and FDR adjusted p-values and Log2 expression means are depicted. Only genes with an adjusted p-value < 0.05 for the comparison between LacQ140 and WT + IPTG are presented to highlight our LacQ140 HD signature. Columns depicting fold changes make the following comparisons: the WT column represents the comparison of WT ± IPTG; the columns LacQ140_A, LacQ140_2M and LacQ140_8M, were each compared to LacQ140; the LacQ140 column compares LacQ140 to WT + IPTG.

Supplementary Table 4. List of individual gene's probability of reversing LacQ140 HD signature transcriptional dysregulation by m*Htt* lowering. Posterior probability reversal values and assignments to probability classes for all signature genes in each of the m*Htt* lowering groups. Each m*Htt* lowering comparison is represented in its own tab. The "Column Definitions" tab describes the column headers used in each tab.

Supplementary Table 5. Overlap of LacQ140 HD signature striatal transcriptional dysregulation compared to a reported multi-HD mouse model striatal dysregulation signature. Multi HD Striatal dysregulation tab: 266 striatal genes have been reported to be consistently dysregulated across multiple HD mouse models(3). Columns B-E report gene name and direction of transcriptional change, based on DEG, and the minimum Log2 fold change across multiple experiments described in (36). Columns G-L: for each of the 266 genes, we report the DEG Log2 fold change and adjusted p-value in 6 and 12 months-old LacQ140, compared to WT + IPTG. In the Significant columns, 1 indicates statistical dysregulation, 0 means not significant (fold change of at least 1.2 and an adjusted p-value < 0.05). Phenotype Reversal tab: Presents the DEG Log2 fold changes of LacQ140_A, LacQ140_2M and LacQ140_8M compared to LacQ140 in the striatum at 6 and 12 months of age. In the Reversal columns, 1 indicates a statistical fold change in the opposite direction of the disease signature, 0 means not significant (adjusted p-value < 0.05). Reversal Summary tab: A summary of the percentage of LacQ140 HD signature transcriptional dysregulation that was reversed with each m*Htt* lowering group.
